# Rice DWARF AND LOW-TILLERING and the homeodomain protein OSH15 interact to regulate internode elongation via orchestrating brassinosteroid signaling and metabolism

**DOI:** 10.1093/plcell/koac196

**Published:** 2022-07-05

**Authors:** Mei Niu, Hongru Wang, Wenchao Yin, Wenjing Meng, Yunhua Xiao, Dapu Liu, Xiaoxing Zhang, Nana Dong, Jihong Liu, Yanzhao Yang, Fan Zhang, Chengcai Chu, Hongning Tong

**Affiliations:** National Key Facility for Crop Gene Resources and Genetic Improvement, Institute of Crop Sciences, Chinese Academy of Agricultural Sciences, Beijing 100081, China; State Key Laboratory of Plant Genomics and Center for Plant Gene Research (Beijing), Institute of Genetics and Developmental Biology, Chinese Academy of Sciences, Beijing 100101, China; National Key Facility for Crop Gene Resources and Genetic Improvement, Institute of Crop Sciences, Chinese Academy of Agricultural Sciences, Beijing 100081, China; National Key Facility for Crop Gene Resources and Genetic Improvement, Institute of Crop Sciences, Chinese Academy of Agricultural Sciences, Beijing 100081, China; Southern Regional Collaborative Innovation Center for Grain and Oil Crops in China, Hunan Agricultural University, Changsha 410128, China; National Key Facility for Crop Gene Resources and Genetic Improvement, Institute of Crop Sciences, Chinese Academy of Agricultural Sciences, Beijing 100081, China; National Key Facility for Crop Gene Resources and Genetic Improvement, Institute of Crop Sciences, Chinese Academy of Agricultural Sciences, Beijing 100081, China; National Key Facility for Crop Gene Resources and Genetic Improvement, Institute of Crop Sciences, Chinese Academy of Agricultural Sciences, Beijing 100081, China; National Key Facility for Crop Gene Resources and Genetic Improvement, Institute of Crop Sciences, Chinese Academy of Agricultural Sciences, Beijing 100081, China; National Key Facility for Crop Gene Resources and Genetic Improvement, Institute of Crop Sciences, Chinese Academy of Agricultural Sciences, Beijing 100081, China; National Key Facility for Crop Gene Resources and Genetic Improvement, Institute of Crop Sciences, Chinese Academy of Agricultural Sciences, Beijing 100081, China; State Key Laboratory of Plant Genomics and Center for Plant Gene Research (Beijing), Institute of Genetics and Developmental Biology, Chinese Academy of Sciences, Beijing 100101, China; National Key Facility for Crop Gene Resources and Genetic Improvement, Institute of Crop Sciences, Chinese Academy of Agricultural Sciences, Beijing 100081, China; National Nanfan Research Institute, Chinese Academy of Agricultural Sciences, Sanya 572024, China

## Abstract

Brassinosteroid (BR) phytohormones play crucial roles in regulating internode elongation in rice (*Oryza sativa*). However, the underlying mechanism remains largely unclear. The *dwarf and low-tillering* (*dlt*) mutant is a mild BR-signaling-defective mutant. Here, we identify two *dlt* enhancers that show more severe shortening of the lower internodes compared to the uppermost internode (IN1). Both mutants carry alleles of *ORYZA SATIVA HOMEOBOX 15* (*OSH15*), the founding gene for *dwarf6*-type mutants, which have shortened lower internodes but not IN1. Consistent with the mutant phenotype, *OSH15* expression is much stronger in lower internodes, particularly in IN2, than IN1. The *osh15* single mutants have impaired BR sensitivity accompanied by enhanced BR synthesis in seedlings. DLT physically interacts with OSH15 to co-regulate many genes in seedlings and internodes. OSH15 targets and promotes the expression of the BR receptor gene *BR INSENSITIVE1* (*OsBRI1*), and DLT facilitates this regulation in a dosage-dependent manner. In *osh15*, *dlt*, and *osh15 dlt*, BR levels are higher in seedlings and panicles, but unexpectedly lower in internodes compared with the wild-type. Taken together, our results suggest that DLT interacts with OSH15, which functions in the lower internodes, to modulate rice internode elongation via orchestrating BR signaling and metabolism.

## Teaser

DWARF AND LOW-TILLERING interacts with the homeodomain protein OSH15, which directly targets the brassinosteroid receptor gene *OsBRI1* and is expressed in lower internodes, to regulate the internode elongation via modulating brassinosteroid signaling and metabolism.

IN A NUTSHELL **Background:** Rice culms consist of five to seven internodes and the length of these internodes determines plant height and resistance to wind, which is crucial for field performance. Brassinosteroid (BR) plant hormones are involved in regulating plant height because defects in BR synthesis or signaling (such as mutants in the BR receptor gene *BRASSINOSTEROID INSENSITIVE 1* (*OsBRI1*)) usually result in dwarfism with specific shortening of the lower internodes or the second internode (IN2) compared to that of the uppermost/first internode (IN1). This pattern is known as *d6* or *dm*-type dwarfism. **Question:** We wanted to know how BRs are involved in organizing the different internodes and therefore, we carried out a large-scale screen for mutants with altered internode organization pattern using the mild BR signaling-defective mutant *dwarf and low-tillering* (*dlt*).

**Findings:** We identified two mutants showing specific shortening of the lower internodes, that is *d6*-type dwarfism. Both mutants have the same causal gene, namely, *OSH15*, which encodes a homeodomain-containing protein. OSH15 can directly interact with DLT, forming a protein complex to regulate BR contents and BR signaling. For example, DLT–OSH15 directly binds the promoter of *OsBRI1* to promote gene expression. *OSH15* expression is strong in the lower internodes, particularly in IN2, and *DLT* shows an opposite expression pattern. Therefore, the protein complex has different levels in different internodes, exerting different effects on BR levels and signaling to modulate internode organization.

**Next steps:** Scientists aim to use BR-related genes to engineer plant height and grain size and thus produce new crops having improved grain yield and lodging resistance. The discovery of the DLT–OSH15–OsBRI1 module could help achieve this goal. Next, we will try to uncover how BRs coordinate internode elongation with panicle development.

## Introduction

Stem elongation is crucial for the development and organization of other organs, such as leaves and branches, and thus influences whole-plant architecture and plant biomass. Culm length (or plant height) is a key agronomic trait affecting lodging resistance in crops. The Green Revolution of the 20th century demonstrated the great success that results from breeding dwarf plant types. However, developing plants of optimal height that balance yield and lodging resistance remains a prime breeding target for most crops, including rice (*Oryza sativa*), maize (*Zea mays*), and wheat (*Triticum aestivum*).

Phytohormones such as gibberellic acids (GAs) and brassinosteroids (BRs) are important determinants of plant height. Several *dwarf* (*d*) mutants, such as *d1*, *d2*, *d11*, *d18*, and *d61*, are defective in GA or BR signaling or biosynthesis (Ueguchi-Tanaka et al., 2000; Yamamuro et al., 2000; Hong et al., 2003; Sakamoto et al., 2004; Tanabe et al., 2005). The genes underlying the favorable traits in the Green Revolution in both rice and wheat are GA-related genes (Peng et al., 1999; Sasaki et al., 2002). Both the hormones and their inhibitors could be used in the field as plant growth modulators (Divi and Krishna, 2009). BRs and GAs extensively interact to regulate cell elongation (Bai et al., 2012; Gallego-Bartolome et al., 2012; Li et al., 2012; Tong et al., 2014; Unterholzner et al., 2015). In addition to plant height, BRs and GAs regulate many other biological processes (Tong and Chu, 2016). For example, BRs strongly promote leaf bending and grain size, whereas GAs play a predominant role in seed germination and flowering.

The rice culm consists of nodes and internodes. Most rice cultivars produce four to seven elongated internodes (counted from top to bottom so that the uppermost internode is labeled as IN1). Internodes begin to develop during the adult phase of the vegetative stage of rice and start to elongate at the reproductive stage (termed the jointing stage; Itoh et al., 2005). Prior to flowering, these internodes elongate sequentially in conjunction with the development and differentiation of young panicles. However, the details of this process have not been fully described. In most cases, the lower internodes elongate first, followed by the upper internodes. The elongation processes of different internodes usually overlap and are coordinated with each other, particularly during panicle development. In general, elongation of the lower internodes corresponds to the booting stage and that of the upper internodes corresponds to the heading stage. How this coordinated process is regulated remains largely unclear.

Numerous dwarf mutants have been isolated in rice. These mutants are classified into six types according to the shortening pattern of the internodes (Yamamuro et al., 2000). The *d6*-type mutants show shortening of all lower internodes except IN1, whereas those of the *dm* type show specific shortening of second internode (IN2). The founding mutants of *d6*-type dwarfism carry defective alleles of *ORYZA SATIVA HOMEOBOX 15* (*OSH15*), a knotted1-like homeobox gene (Sato et al., 1999). In addition, *dm* and *d6* types of dwarfism have been observed in a number of BR-defective plants (Yamamuro et al., 2000; Tanabe et al., 2005). Intriguingly, severe BR-defective plants, such as *d61-2* and *Go-2*, tend to show *d6*-type dwarfism (Yamamuro et al., 2000; Tong et al., 2012), whereas mild mutants, such as *d11* and *d61-1*, tend to show *dm*-type dwarfism (Yamamuro et al., 2000; Tanabe et al., 2005). Clearly, BRs play a critical role in regulating the differential elongation of internodes, particularly IN2. The expression of *BR INSENSITIVE 1* (*OsBRI1*), encoding a BR receptor, is specifically reduced in IN2 and IN3 compared to the other internodes, suggesting that the specific shortening of IN2 might be attributed to differential sensitivity to BRs in different internodes (Yamamuro et al., 2000).

We previously identified the semidwarf mutant *dwarf and low-tillering* (*dlt*), which shows a mild BR-signaling-deficient phenotype (Tong et al., 2009). Compared to the wild-type, all of the internodes in *dlt* are shortened, but IN2 tends to be shortened more markedly than the others (Tong et al., 2009). Therefore, *dlt* is suitable for the genetic screening of additional regulators involved in internode elongation processes modulated by BRs. Indeed, by performing large-scale mutagenesis and screening, we identified a number of *dlt* enhancers showing *d6*- or *dm*-type dwarfism. Here, we describe two of these enhancers in detail and demonstrate that DLT interacts with OSH15 to regulate the elongation of rice internodes, particularly the lower ones, via modulating BR responses.

## Results

### Identification of *dlt* enhancers showing *d6*-type dwarfism

**Figure 1:**
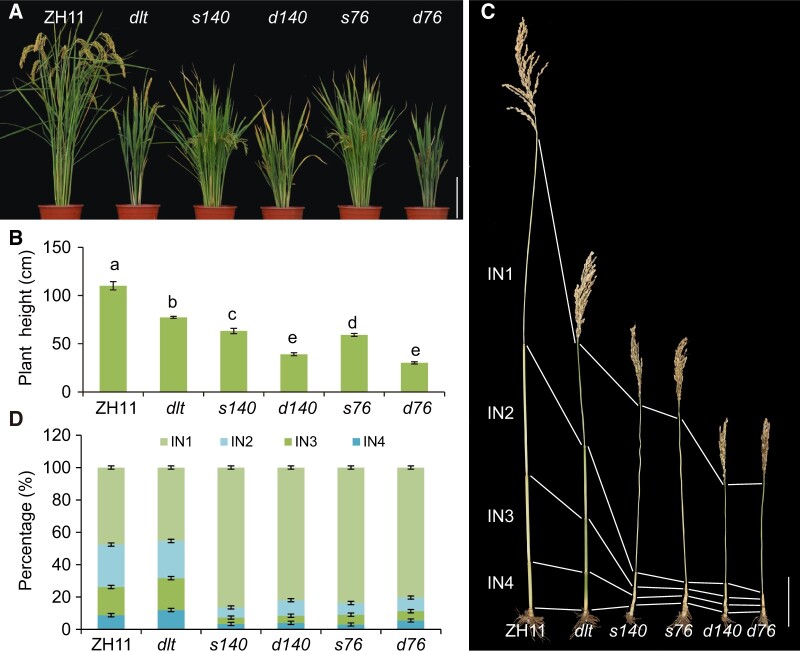
Isolation of two *dlt* enhancers showing *d6*-type dwarfism. A, Morphology of ZH11, *dlt*, *s140*, *d140*, *s76*, and *d76* plants at 105 days after sowing. Bar = 20 cm. The *d* prefix indicates double mutants with *dlt* and the *s* prefix indicates single mutants in the wild-type *DLT* background. B, Statistical analysis of the height of the plants shown in (A). Data are shown as means ± sd (*n* = 15). Different letters on the histograms indicate statistically significant differences at *P* < 0.05 by pairwise multiple comparison followed with Tukey’s test. C, Culm structures of the plants at 145 days after sowing. IN, internode. Bar = 10 cm. D, The percentages of the lengths of different internodes in different plants. Data are shown as means ± sd (*n* = 15).

We have generated a mutant library containing approximately 25,000 lines by mutagenesis of *dlt* seeds using sodium azide as mutagen (Liu et al., 2022). Screening of the library led to the identification of a number of *dlt* enhancers displaying further reduced plant height (Figure 1, A and B). To identify additional genes involved in the BR-mediated control of internode elongation, we screened for *dlt* enhancers showing shortening of a specific internode for further analysis. Among these, *d76* and *d140* (*d* for *dlt*-based double mutant) exhibited a typical *d6*-type dwarfism pattern (Figure 1, C and D). We backcrossed the mutants with wild-type Zhonghua11 (ZH11, a *japonica* cultivar) for genetic analysis.

Among the 120 F_2_ progeny of the *d140 *×* *ZH11 cross, 29 plants showed *d6*-type dwarfism, whereas the others showed *dlt* or wild-type phenotypes. The segregation ratio (29:91) was close to 1:3 (χ^2^ = 0.011), suggesting that *d6*-type dwarfism is controlled by a recessive mutation. In addition, 24 plants from the F_2_ population showed an intermediate phenotype between *dlt* and *d140*; these plants were likely single mutants only containing the new mutation. After backcrossing these mutants with the wild-type for at least two additional generations, a single mutant, designated *s140*, was obtained and used for further analysis. Similar results were obtained for *d76*, and *s76* was generated as well. Compared to the respective double mutants, both single mutants showed increased plant height (Figure 1, A–D). However, similar to the respective double mutants, *s76* and *s140* still showed *d6*-type dwarfism, with comparable levels of severity in terms of internode length (Figure 1, C and D).

### *s76* and *s140* are new alleles of *d6*/*osh15*, the founding mutant used to define *d6*-type dwarfism

**Figure 2:**
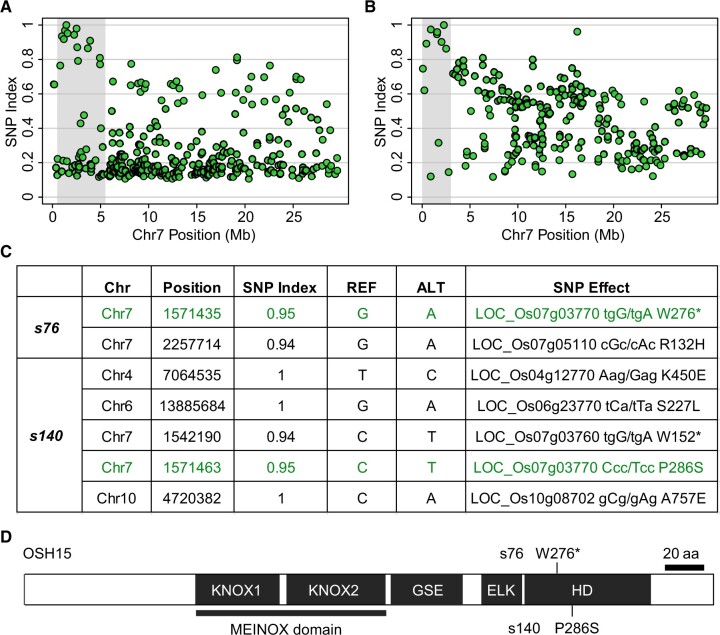
Cloning of the mutant genes using the MutMap strategy. A, B, SNP index plots for *s76* (A) and *s140* (B) on chromosome 7. Each dot on the plot represents a mutation and its corresponding allele frequency (SNP index) in the mutant pool. The peak regions for candidate mutations (shaded in gray) have the highest SNP indices at potential causal SNPs and decreasing SNP indices for mutations in more distant regions, a pattern expected under recombination. C, Information about the candidate mutations (SNPs), genes, and effects of the mutations on the codons. Coloured text indicates shared candidates between the two mutants. D, Mutation information of *s76* and *s140* in OSH15 protein. Schematic diagram of OSH15 protein structure was shown, with the KNOX1, KNOX2, GSE, ELK, and HD (homeodomain) domains indicated according to a previous report (Nagasaki et al., 2001).

To identify the genomic mutation(s) underlying the dwarf phenotypes of *s76* and *s140*, we utilized the MutMap mapping strategy as described previously (Abe et al., 2012; Yan et al., 2016). We screened F_2_ populations from the backcrosses of *s76 *×* *ZH11 and *s140 *×* *ZH11 and pooled 18 and 76 mutant individuals, respectively, for high-coverage genome sequencing (∼20×). After quality control and reads mapping to the rice reference genome, we screened across the genome to identify mutations specific to the mutant pool, that is those for which the single-nucleotide polymorphism (SNP) index is close to 1 (Supplemental Figures S1 and S2). We also included the other mutant pools for cross-exclusion of background mutations based on the assumption that each mutant should carry a unique mutation spectrum. In both mutant pools, we identified typical association peaks on the target mutation region at similar positions on chromosome 7 (Figure 2, A and B). We searched for functional mutations within the peak regions and identified the *OSH15* gene as the candidate gene for both mutants (Figure 2C). The candidate causal mutations occurred in the homeodomain of OSH15 (Nagasaki et al., 2001) for both mutants. The *s76* mutant carries a G-to-A mutation at amino acid W276, which results in a premature stop codon, while *s140* contains a C-to-T mutation resulting in a P286S substitution (Figure 2, C and D). Given the highly similar phenotypes of the *s76* and *s140* mutants and those reported for *d6*/*osh15*, we concluded that *s76* and *s140* are new alleles of *d6*/*osh15*, the founding mutant of *d6*-type dwarfism.

### OSH15 and DLT cooperatively regulate internode elongation to coordinate with panicle development

**Figure 3:**
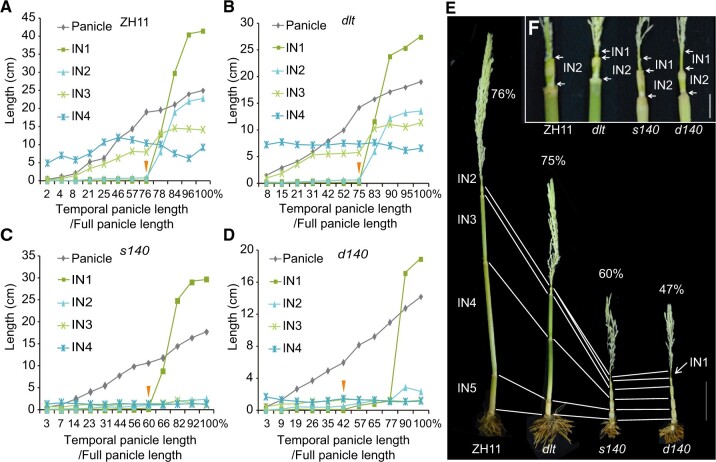
*s140* and *d140* show altered internode elongation. A–D, Elongation patterns of the internodes in coordination with panicle development in ZH11 (A), *dlt* (B), *s140* (C), and *d140* (D). The *d* prefix indicates double mutants with *dlt* and the *s* prefix indicates single mutants in the wild-type *DLT* background. For each mutant or the wild-type, the lengths of the different internodes and panicles in different individuals or tillers were repeatedly measured until development was complete. As panicle development was used as a reference, the percentage of temporal panicle length versus fully developed panicle length per plant (on the abscissa) indicates the developmental point in various tissues. Note that since it was difficult to obtain absolutely the same data points (especially during the rapid elongation stage), the values at representative points were used for plotting. Triangles mark the starting points of IN1 elongation in different plants. E, Comparison of culm organization at or around the beginning of IN1 elongation. Elongation of IN1 was clearly observed in *s140* and *d140* but was not obvious in ZH11 or *dlt*. The percentages of panicle development in each plant are indicated. Bar = 5 cm. F, Magnified views of IN1 and IN2 in (E). Bar = 1 cm.

It is unclear how the panicle and internodes, which exhibit intertwined development in the rice culm, are coordinately elongated. Assuming that internode elongation is always coordinated with panicle development, we used panicle development (temporal panicle length/full panicle length) as an internal reference (proxy for time points) to evaluate internode elongation to avoid the interference of developmental differences among different tillers or plants. In ZH11 and *dlt*, IN4 had already elongated when the panicle started to develop (Figure 3, A and B). IN3 began to elongate at exactly the same time; this elongation appeared to be synchronized with panicle development (Figure 3, A and B). Elongation of IN2 and IN1 occurred at the same time point in ZH11 and *dlt* and was only observed when the panicle developed to ∼75% of its full length (Figure 3, A and B). However, in *s140* and *d140*, elongation of IN1 occurred earlier, that is when the panicle developed to ∼60% and ∼42% of its full length, respectively (Figure 3, C–F). Therefore, IN1 elongation appeared to occur earlier in *d140* than in *s140*, suggesting that DLT likely interacts with OSH15 to regulate this process. Interestingly, although IN2 was short in both *s140* and *d140*, its elongation was also observed at or around this time (Figure 3, C and D). Thus, OSH15 might prevent the early elongation of IN1 and IN2 to coordinate with panicle development. Notably, the elongation of IN1 in *d140* occurred slowly at the beginning (42%–77%), in contrast to IN1 in *dlt* and *s140*, which elongated rapidly at this stage, suggesting that DLT and OSH15 have synergistic effects in controlling the IN1 elongation rate.

### Expression of *OSH15* and *DLT* in different internodes

**Figure 4:**
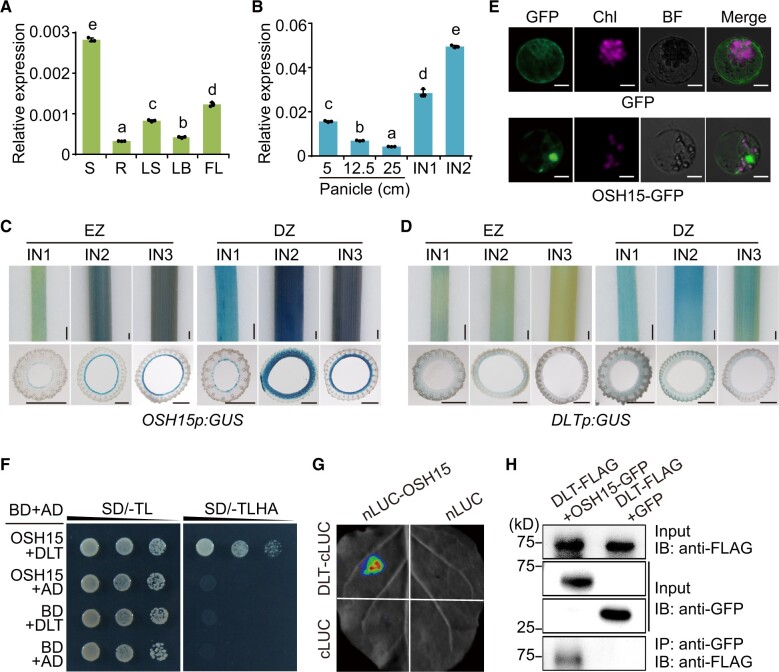
Expression specificities and interaction between DLT and OSH15. A, Relative expression levels of *OSH15* in various tissues, including seedling shoot (S), root (R), young leaf sheath (LS), young leaf blade (LB), and flag leaf blade (FL). Data are shown as means ± sd (*n* = 3). Different letters on the histograms indicate statistically significant differences at *P* < 0.05 by pairwise multiple comparison followed by Tukey’s test. B, Relative expression levels of *OSH15* in panicles in different length and internodes. Data are shown as means ± sd (*n* = 3). Different letters on the histograms indicate statistically significant differences at *P* < 0.05 by pairwise multiple comparison followed by Tukey’s test. C, D, Evaluation of the promoter activity in different internodes by GUS staining. EZ, elongating zone; DZ, division zone. Transverse sections for each part were shown below the internodes. Bar = 1.5 mm. E, Subcellular localization of OSH15-GFP in rice protoplasts. GFP was expressed as a control using empty vector. BF, bright field; Chl, chlorophyll autofluorescence. Bar = 10 mm. F, Interaction between DLT and OSH15 in yeast two-hybrid assay. SD/-TL, selective medium lacking Trp and Leu. SD/-TLHA, lacking Trp, Leu, His, and Ade. G, Split-luciferase complementation analysis of the interaction between OSH15 and DLT. H, Co-IP analysis of the interaction between OSH15–GFP and DLT–FLAG. IB, immunoblotting analysis. Marker size was indicated.

Consistent with previous reports (Sato et al., 1999; Yoon et al., 2017), *OSH15* was expressed at relatively low levels in seedling tissues (Figure 4A), but was strongly expressed in panicles and internodes, as revealed by reverse transcription–quantitative polymerase chain reaction analysis (RT–qPCR; Figure 4B). Notably, in internodes, *OSH15* expression was higher in IN2 compared to IN1 (Figure 4B).

To confirm this result, we produced transgenic plants harboring the β-glucuronidase (GUS) reporter driven by *OSH15* promoter and *DLT* promoter, respectively. Strikingly, GUS staining of different internodes revealed that the *OSH15* promoter had the strongest activity in IN2, particularly in the lower part corresponding to the division zone (Figure 4C). The staining in division zone of IN1 was also clear but much weaker compared to that of IN2 or IN3 (Figure 4C). In elongating zone, the staining gradually increased from IN1 to IN3 (Figure 4C). Transverse sections revealed that OSH15 was specifically expressed in the parenchyma cells of most materials except the division zone of IN2 where an extended expression area was detected (Figure 4C).

In contrast, the *DLT* promoter overall showed relatively mild activity (Figure 4D). However, in the elongating zone as well as the division zone, the staining shade gradually decreased from IN1 to IN3 (Figure 4D). This expression tendency is consistent with the previous expression analysis (Tong et al., 2009), but is opposite to the pattern of *OSH15*. Transverse sections revealed that the staining appeared to be specific in the parenchyma cells of the materials having relatively low expression levels (Figure 4D). These analyses suggested that *DLT* and *OSH15* could have overlapping expression territories but opposite expression specificities in different internodes.

### DLT interacts with OSH15

To examine the subcellular localization of OSH15, we expressed an OSH15-green fluorescent protein (GFP) fusion in protoplasts. The OSH15–GFP fusion protein was present in both the cytoplasm and nuclei of transgenic rice protoplasts, with more pronounced signals in the nuclei, where OSH15 showed clear colocalization with DLT (Figure 4E;Supplemental Figure S3).

Yeast two-hybrid analysis showed that DLT interacts with OSH15 and that the C-terminus of DLT (containing a GRAS domain) is responsible for this interaction (Figure 4F;Supplemental Figure S4). OSH15 can also interact with itself, but unlike DLT, it cannot interact with GLYCOGEN SYNTHASE KINASE2 (GSK2) kinase, the key negative regulator of BR signaling (Supplemental Figure S4; Tong et al., 2012). We confirmed the interaction between DLT and OSH15 by split-luciferase complementation analysis conducted in *Nicotiana benthamiana* leaves (Figure 4G). Coimmunoprecipitation (Co-IP) analysis using GFP/FLAG-tagged fusion proteins further confirmed the interaction between these proteins (Figure 4H). These results strongly suggest that DLT and OSH15 form a complex to regulate downstream biological processes.

### OSH15 participates in BR responses

**Figure 5:**
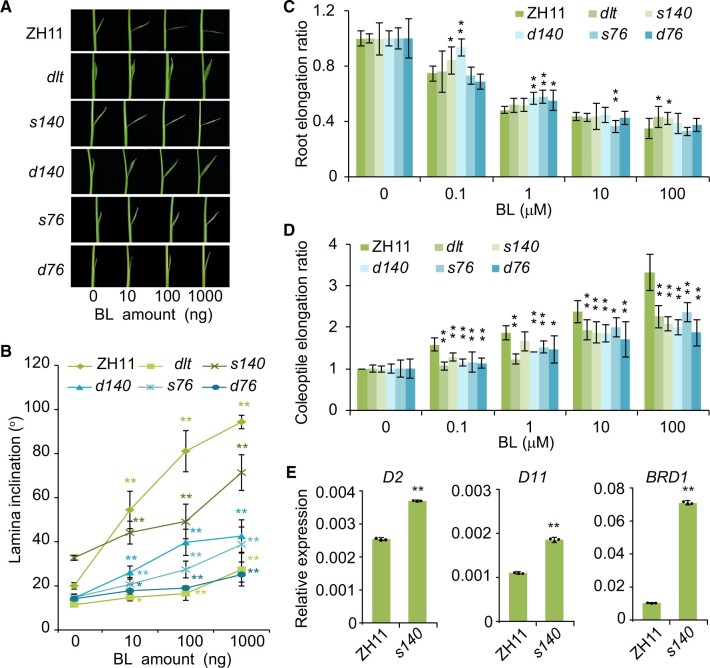
OSH15 regulates BR responses. A, Lamina bending analysis of ZH11, *dlt*, *s140*, *d140*, *s76*, and *d76* in response to BL. The *d* prefix indicates double mutants with *dlt* and the *s* prefix indicates single mutants in the wild-type *DLT* background. B–D, Quantification of the leaf angles (B) shown in (A). Root (C) and coleoptile (D) elongation assay in response to BL. Data are shown as means ± sd (*n* = 8). Asterisks indicate significant difference compared with the sample without BL, with *P < 0.05 and **P < 0.01 by Student’s two-sided *t* test. E, Relative expression levels of the three BR biosynthetic genes in ZH11 and *s140*. Fifteen-day-old seedling shoots were used for RT-qPCR analysis. Data are shown as means ± sd (*n* = 3). Asterisks indicate significant difference compared with ZH11, with **P < 0.01 by Student’s two-sided *t* test.

Typical BR signaling mutants, such as *dlt* and *d61*, usually show reduced sensitivity to BR but increased expression of BR biosynthetic genes due to feedback regulation (Yamamuro et al., 2000; Tong et al., 2009). A lamina inclination assay revealed that, like *dlt*, both *s76* and *s140*, as well as *d76* and *d140*, were less sensitive to treatment with the active BR brassinolide (BL) than the wild-type (Figure 5, A and B). We also performed BR sensitivity tests in root and coleoptile. In root analysis, both *s76* and *s140*, as well as *d76* and *d140*, had only slight or basically no alteration in response to BL treatment (Figure 5C). However, in coleoptile, all the mutants indeed showed decreased BR sensitivity (Figure 5D). These results are consistent with our previous report that *dlt* is less sensitive to BR in lamina joints and coleoptiles, but shows normal sensitivity to BR in root (Tong et al., 2009).

Interestingly, in the absence of BR treatment, *s140*, but not *s76*, actually showed slightly increased leaf angles compared to the wild-type (Figure 5, A and B), perhaps due to the increased BR levels in the mutant (Supplemental Table S1; see subsequently). Indeed, the BR biosynthetic genes *D2*, *D11*, and *BRD1* showed increased expression in *s140* versus the wild-type (Figure 5E). These results indicated that OSH15 also participates in BR responses and likely interacts with DLT to regulate BR signaling and biosynthesis. In addition, *d140* has a partially rescued BR sensitivity compared to *dlt*. Considering that BR levels were greatly enhanced in *d140* (Supplemental Table S1), one possibility is that the highly accumulated BRs in *d140* have activated other branched lamina bending pathways such as the BRASSINAZOLE RESISTANT1 (BZR1) pathway (Tong and Chu, 2018).

### DLT and OSH15 exhibit a tissue-dependent genetic relationship

**Figure 6:**
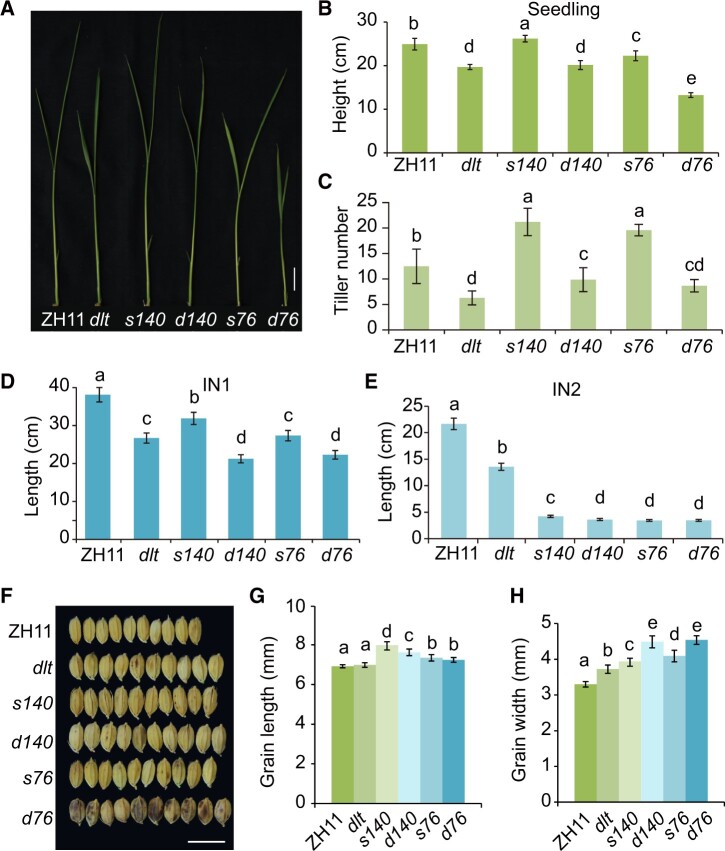
DLT and OSH15 exhibit a variable genetic relationship in different tissues. A, Seedling morphology of ZH11, *dlt*, *s140*, *d140*, *s76*, and *d76* at 15 days after germination. Bar = 2 cm. The *d* prefix indicates double mutants with *dlt* and the *s* prefix indicates single mutants in the wild-type *DLT* background. B, Statistical analysis of seedling length in (A). Data are shown as means ± sd (*n* = 13). Different letters on the histograms indicate statistically significant differences at *P* < 0.05 by pairwise multiple comparison followed with Tukey’s test. C–E, Statistical analysis of tiller number (C) and IN1 (D) and IN2 length (E) in ZH11, *dlt*, *s140*, *d140*, *s76*, and *d76* at 105 days after sowing. Data are shown as means ± sd (*n* = 12). Different letters on the histograms indicate statistically significant differences at *P* < 0.05 by pairwise multiple comparison followed with Tukey’s test. F, Comparison of the width of 10 grains of ZH11, *dlt*, *s140*, *d140*, *s76*, and *d76*. Bar = 10 mm. G, H, Statistical analysis of grain length (G) and grain width (H) of ZH11, *dlt*, *s140*, *d140*, *s76*, and *d76*. Data are shown as means ± sd (*n* = 16). Different letters on the histograms indicate statistically significant differences at *P* < 0.05 by pairwise multiple comparison followed with Tukey’s test.

Both *s76* and *s140* showed weak phenotypes at the seedling stage (Figure 6A). The seedling height of *s140* was slightly greater than that of the wild-type (5.17% increase), whereas the seedling height of *s76* was slightly reduced (10.70% decrease; Figure 6B;Supplemental Table S2). While the seedling heights of *dlt* and *d140* showed similar levels of reduction compared to the wild-type (20.96% and 19.20%, respectively), the seedling height of *d76* was much more strongly reduced (46.80% decrease; Figure 6, A and B; Supplemental Table S2). Given that *s76* carries a stronger mutation in OSH15 than *s140*, these results suggest that DLT functions synergistically with OSH15 to regulate seedling height. In contrast to *dlt*, which had fewer tillers than the wild-type, *s76* and *s140* developed many more tillers than the wild-type, whereas *d76* and *d140* had slightly fewer (Figure 6C), suggesting that DLT and OSH15 have antagonistic effects in controlling tiller number.

Both *d76/d140* and *s76/s140* showed *d6*-type dwarfism. The culm length of *dlt* was ∼70.2% that of the wild-type, whereas the culm length of *d76* was ∼78.3% that of *s76*, and the culm length of *d140* was ∼73.6% that of *s140* (Supplemental Table S2). The results suggest that DLT partially requires OSH15 to regulate culm length. In detail, the length of IN2 was similar in the double mutants (*d76* and *d140*) and the single mutants (*s76* and *s140*), whereas IN1 was shorter in *d76* and *d140* than in *s76* and *s140* (Figure 6, D and E; Supplemental Table S2). These findings suggest that OSH15 is epistatic over DLT in regulating the elongation of IN2 but has an additive effect with DLT in regulating the elongation of IN1. In the transverse direction of the culm, cell number increased in *dlt* but decreased in *s140* compared to the wild-type, whereas it was barely changed in *d140* (Supplemental Figure S5), suggesting that DLT and OSH15 have antagonistic effects in regulating the transverse growth of internode cells.

Intriguingly, the grain sizes of *s76* and *s140* were obviously increased in terms of both length and width (Figure 6, F–H). The grain width was strongly increased in *dlt* and further increased in *d76* and *d140* (Figure 6, F and H), suggesting that DLT and OSH15 have additive effects in regulating grain width. These findings suggest a tissue-dependent genetic relationship between OSH15 and DLT.

### DLT cooperates with OSH15 to activate common genes in seedlings

Both OSH15 and DLT are putative transcription factors, and OSH15 has been shown to have transcription activation activity in yeast as well as DNA-binding activity in vitro (Nagasaki et al., 2001). To assess their functions at the molecular level, we performed transcriptome analysis using the shoots of mutant (*dlt*, *s140*, and *d140*) and wild-type (ZH11) seedlings since the tissues are most feasible to sample. We identified differentially expressed genes (DEGs) in the three mutants compared to ZH11. Specifically, we identified 1,128 down- and 217 upregulated DEGs in *dlt*, 866 down- and 348 upregulated DEGs in *s140*, and 1,853 down- and 213 upregulated DEGs in *d140* (Supplemental Data Set S1). The high ratios of downregulated genes to upregulated genes indicate that both OSH15 and DLT could function as transcriptional activators.

**Figure 7:**
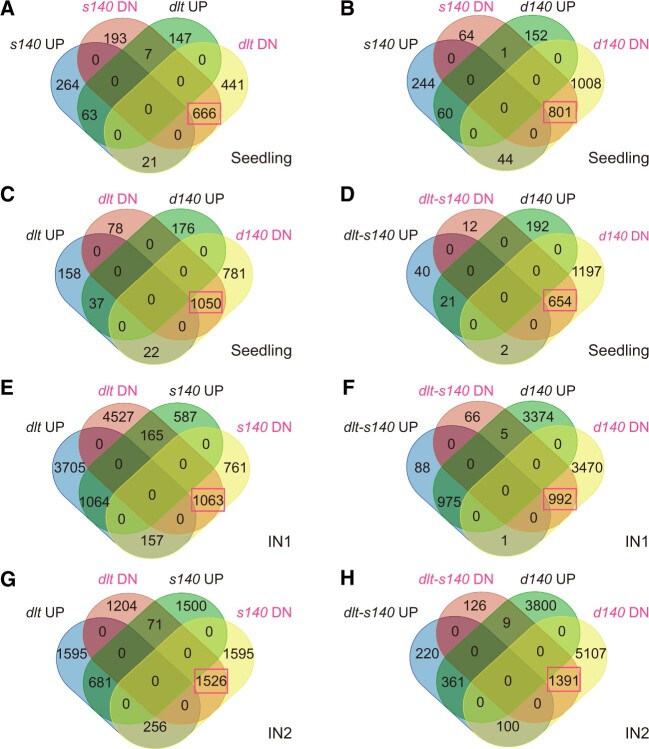
DLT and OSH15 coregulate gene expression in various tissues. DEGs were identified in different tissues of the mutants via comparison with the corresponding tissues of the wild-type and subjected to overlapping analysis. Data are shown as Venn diagrams to compare the number of overlapping DEGs between sample sets, as indicated in the sectors. UP, upregulated DEGs in a mutant compared to the wild-type. DN, downregulated DEGs. The number of overlapping downregulated DEGs in different sample sets are highlighted with boxes.

Of these DEGs, 757 were identified in both *s140* and *dlt*, accounting for 62.4% of the DEGs in *s140* (757/1,214) and 56.3% in *dlt* (757/1,345; Figure 7A). Importantly, of these overlapping DEGs, 96.3% (729/757) were regulated in the same manner by OSH15 and DLT, with 91.4% (666/729) downregulated in both *s140* and *dlt* (Figure 7A). In addition, most downregulated DEGs in *dlt* (93.1%, 1,050/1,128) or *s140* (92.5%, 801/866) were also downregulated in *d140* (Figure 7, B and C). Of the 666 downregulated DEGs in both *dlt* and *s140*, 98.2% (654/666) were also downregulated in *d140* (Figure 7D). Most of these genes (61.9%, 405/654) were expressed at lower levels in *d140* than in either single mutant.

Moreover, 90.1% (1,197/1,328) of the genes that were downregulated in *dlt* or *s140* were also differentially regulated in *d140*. Conversely, 656 DEGs were downregulated only in the double mutant and not in any single mutant (Figure 7D). The expression of these additional genes was suppressed due to the simultaneous loss of function of DLT and OSH15. The co-downregulated DEGs in all the three mutants (Supplemental Data Set S1) were further used for cluster analyses, and a high similarity of the expression profiles between *d140* and *dlt* was observed, whereas *s140* exhibited much lower similarity with both *d140* and *dlt* (Supplemental Figure S6A). These findings suggest that DLT and OSH15 cooperate with each other, with DLT playing a more dominant role in activating gene expression in seedlings.

### DLT and OSH15 cooperate to regulate gene expression in internodes

We performed similar transcriptome analyses using IN1 and IN2. In IN1, we identified 5,755 down- and 4,926 upregulated DEGs in *dlt*, 1,981 down- and 1,816 upregulated DEGs in *s140*, and 4,463 down- and 4,354 upregulated DEGs in *d140* (Figure 7, E and F; Supplemental Data Set S2). In IN2, we identified 2,801 down- and 2,532 upregulated DEGs in *dlt*, 3,377 down- and 2,252 upregulated DEGs in *s140*, and 6,598 down- and 4,170 upregulated DEGs in *d140* (Figure 7, G and H; Supplemental Data Set S3).

First, in all mutants, the downregulated DEGs outnumbered the upregulated DEGs in both internodes, although to a much smaller extent than in seedlings. Second, *dlt* and *s140* shared many DEGs in both internodes, most of which had consistent expression patterns (up- or downregulated; Figure 7, E–H). Third, most of these shared DEGs in the two single mutants were also differentially regulated in the double mutant, with similar expression patterns (Figure 7, E–H). In general, these observations are highly consistent with the observations in seedlings, demonstrating that DLT and OSH15 cooperate with each other to regulate gene expression in various tissues.

Interestingly, in *dlt* more DEGs were identified in IN1, whereas in *s140* more DEGs were identified in IN2. In addition, more DEGs were identified in IN2 than IN1 of the double mutant. Clustering analysis of the co-downregulated DEGs in the three mutants (Supplemental Data Sets S2 and S3) revealed that, in IN1, *d140* and *dlt* tended to have similar expression profiles, yet the distances in expression patterns among the three mutants were actually very close each other (Supplemental Figure S6B). However, in IN2, *dlt* and *s140*, the two single mutants, were most closely associated (Supplemental Figure S6C). Notably, gene expression pattern in *d140* was highly similar to *s140* but not *dlt* (Supplemental Figure S6C). All these results are consistent with the relative expression levels of the two genes, that is *DLT* expression is lower in IN2 than IN1, and *OSH15* has a reversed expression pattern. We noted that the number of DEGs in *d140* is decreased in IN1 compared to *dlt*. One possibility is that OSH15 antagonizes the function of DLT in other aspects, such as culm width in IN1 (Supplemental Figure S5), leading to fewer DEGs in the double mutant.

We could only identify a small number of the co-regulated DEGs between seedling and internodes (IN1 and IN2) in all the mutants. To verify the transcriptome analysis results, we selected ten downregulated DEGs shared in IN1 and IN2 of *d140* for RT–qPCR analyses and obtained results highly consistent with the transcriptome analysis. All of these genes had sharply decreased expression in both IN1 and IN2 of the single and double mutants (Supplemental Figure S7). Compared to either of the single mutants, most of these genes had the lowest expression in the double mutant (Supplemental Figure S7). However, when using seedling for the analyses, none of these genes exhibited a similar expression pattern as in internodes (Supplemental Figure S7). Therefore, we consider the lack of co-regulated DEGs between seedling and internodes could be attributable to either the distinctive transcriptome profiles in the two tissues or the differential regulation of gene expression profiles by OSH15 and DLT in the two tissues, or both.

### DLT and OSH15 coregulate various biological processes and traits

To dissect the function of DLT and OSH15, we conducted both gene ontology (GO) and trait ontology (TO) analyses using either the full DEG list or the downregulated DEGs in IN1 and IN2 of the three mutants. In each mutant, GO analyses identified a number of significant terms (corrected *P* < 0.05) associated with biological pathways (such as “regulation of transcription”), molecular functions (such as “nucleic acid binding”), and cellular component (such as “nucleus”) (Supplemental Data Set S4). Notably, TO analyses revealed that both DLT and OSH15 significantly regulate many genes involved in agronomic traits, such as “plant height”, “stem length”, and “dehulled grain weight” (Supplemental Data Set S5).

For clarification, here we describe the results obtained from the IN2 as an example. When using the downregulated DEGs for the analyses, we identified 31, 55, and 67 significant GO terms in *dlt*, *s140* and *d140* respectively (Supplemental Data Set S4). Many of them are related to regulation of transcription, phytohormone response, and stress response (Supplemental Data Set S4). Interestingly, the term “brassinosteroid mediated signaling pathway” was only identified in *d140*. Eleven biological pathway terms were enriched in all three mutants, such as “regulation of transcription” and “response to salt stress” (Supplemental Figure S8). In TO analyses, 12 terms were identified from all three mutants (Supplemental Figure S9). These terms are strongly related to agronomic traits, such as “plant height” and “panicle length”, or stress response, such as “salt tolerance” and “drought tolerance”. For each term, the DEG number in the double mutant was always more than that in either single mutant. Some of these terms are apparently associated with the mutant phenotypes or the protein functions. Besides, the identification of a number of stress-related GO and TO terms suggested an additional role of DLT–OSH15 module in stress responses (Supplemental Data Sets S4 and S5).

### OSH15 targets a number of BR-related genes

In all three tissues of the three mutants, we identified a number of DEGs involved in BR biosynthesis, catabolism, and signaling (Supplemental Figure S10). To further explore whether OSH15 directly targets BR-related genes, we performed chromatin immunoprecipitation assay followed by sequencing analysis (ChIP-seq) to search for OSH15-binding regions. To this end, we first tried to generate transgenic plants overexpressing OSH15–FLAG fusion proteins in order to utilize the tag antibody for the analysis. However, most of the transplants failed to survive in field due to the severe defect of shoot development (Supplemental Figure S11), similar to previously reported results (Sentoku et al., 2000). Therefore, calli transfected with OSH15–FLAG were used for ChIP instead.

**Figure 8:**
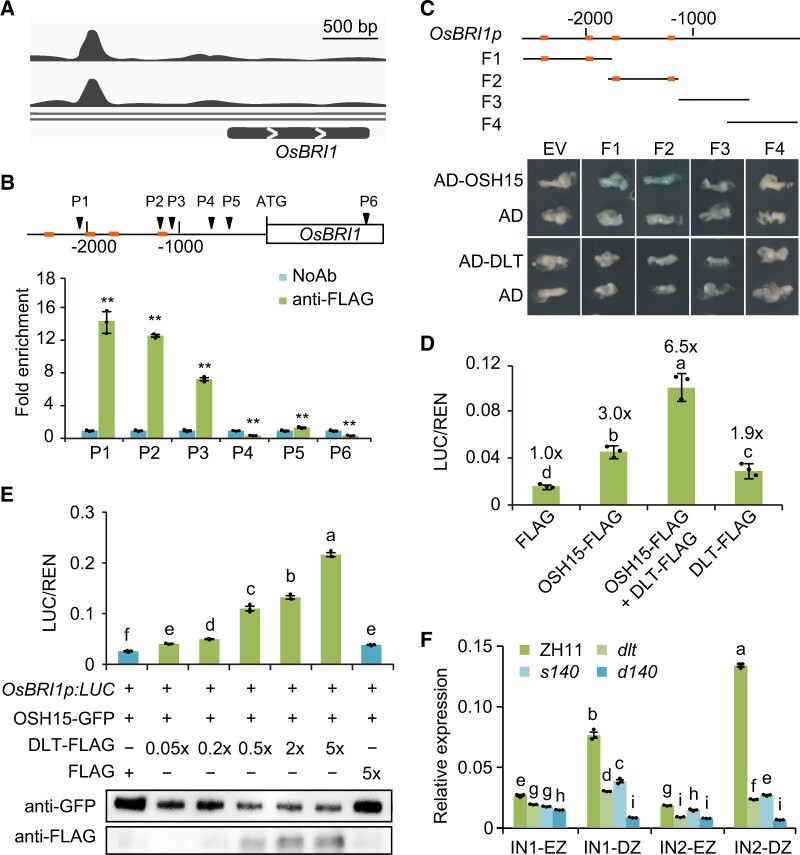
OSH15 targets and promotes *OsBRI1* expression, facilitated by DLT. A, Visualization of ChIP-seq data showing the peaks on *OsBRI1* promoter with two replicates. B, Confirmation of OSH15-binding on *OsBRI1* promoter by ChIP-qPCR. Fold enrichment was shown as ratios of antibody-precipitated samples on those without antibody application (NoAb, set at 1.0). The amplified regions (P1–P6) are marked schematically on *OsBRI1* gene, with the TGTCAC elements indicated (coloured thick bar). Data are shown as means ± sd (*n* = 3). Asterisks indicate significant difference compared with NoAb, with **P < 0.01 by Student’s two-sided *t* test. C, Yeast one-hybrid assay showing the binding of OSH15, but not DLT, on *OsBRI1* promoter. Four fragments of *OsBRI1* promoter (F1–F4) were used for the test, with the distribution of TGTCAC elements indicated on each by coloured thick bars. EV, empty vector. D, E, Effect of OSH15 and DLT on *OsBRI1* promoter activity evaluated by luciferase reporter assay. Data are shown as means ± sd (*n* = 3). The fold changes were marked on each column in (D). The protein levels were tested and shown below the histogram in (E). Different letters on the histograms indicate statistically significant differences at *P* < 0.05 by pairwise multiple comparison followed with Tukey’s test. F, Expression of *OsBRI1* in the elongating zone (EZ) and division zone (DZ) of different internodes in different plants. Data are shown as means ± sd (*n* = 3). Different letters on the histograms indicate statistically significant differences at *P* < 0.05 by pairwise multiple comparison followed with Tukey’s test.

Calli transfected with an empty vector were used as reference. Among a number of genomic peaks bound by OSH15 identified from two biological replicates, we focused on those distributed in 3′-end regions of BR-related genes. As a result, a dozen BR-related genes were clearly associated with OSH15 and the binding pattern is highly consistent in two replicates (Figure 8A;Supplemental Figure S12). These genes include many primary BR signaling genes such as *OsBRI1*, *BR SIGNALING KINASE1-2/2*, *GSK1/3*, and *OsBZR2/3*, and several BR metabolism genes such as *D11* and *OsCYTOCHROME P450 734A5/6*, suggesting that OSH15 might directly regulate both BR homeostasis and BR signaling. Interestingly, *DLT*, *SMALL ORGAN SIZE1 (SMOS1)*, and *OSH1* as well as two additional OSH members (*OSH3* and *OSH71*) were also included. It is known that OSH15 can interact with both DLT and OSH1 (Nagasaki et al., 2001), and DLT can also interact with both SMOS1 and OSH1 (Hirano et al., 2017; Yang et al., 2018). Therefore, these proteins might form a protein complex to function, as has been suggested previously (Xiao et al., 2020). The targeting of OSH15 to these genes suggested that OSH15 might play a pivotal role in regulating the complex constitution by modulating the transcription of its components.

### OSH15 binds the *OsBRI1* promoter region

We selected the *OsBRI1* promoter region for further verification because its associated peak is most specific compared to other peaks (Figure 8A). In addition, it has been shown that plants defective in *OsBRI1* frequently exhibit *dm-* or *d6-*type dwarfism (Yamamuro et al., 2000), strongly suggesting that *OsBRI1* could participate in OSH15-mediated regulation of internode elongation.

Sequence analysis revealed that the *OsBRI1* promoter region (∼2.5 kb) contains four potential OSH15 binding cis-elements (TGTCAC; Nagasaki et al., 2001; Figure 8B), with one close to the peak site (−1.2 kb). We introduced *OSH15–FLAG* as well as the whole *OsBRI1* gene (containing the 2.5-kb promoter) into *N.benthamiana* leaves and then performed ChIP-quantitative PCR (qPCR) analysis. Of six sites detected using the corresponding primers (P1–P6), OSH15 was strongly associated with three of them (P1–P3), corresponding to the positions where the cis-elements reside (Figure 8B). Furthermore, we split the promoter into four fragments (F1–F4) and tested the protein–DNA interaction by yeast one-hybrid assay. As results, OSH15 was able to interact with both F1 (−2,594 to −1,767) and F2 (−1,785 to −1,146), two fragments containing the putative binding elements, but not F3 and F4 (Figure 8C). However, when OSH15 was replaced with DLT, no interaction was detected for all the four fragments (Figure 8C).

### OSH15 activates *OsBRI1* expression and DLT promotes the regulation

Next, we tested the effect of OSH15 binding on *OsBRI1* promoter activity using luciferase as the reporter. The expression of OSH15–FLAG significantly enhanced *OsBRI1* promoter activity, leading to a ∼3-fold increase compared to that expressing only the FLAG tag. DLT–FLAG conferred a similar effect, but to a lesser extent (∼1.9-fold; Figure 8D). Strikingly, when the two proteins were co-expressed, the promoter activity was increased ∼6.5-fold (Figure 8D), suggesting that DLT and OSH15 could promote *OsBRI1* expression in a synergistic manner. Importantly, when DLT–FLAG expression increased in a gradient, the promoter activity increased accordingly, with a maximum ∼10-fold compared to OSH15 alone (Figure 8E).

Consistently, *OsBRI1* expression was markedly decreased in both IN1 and IN2 of all the mutants compared with the wild-type (Figure 8F). Notably, the decrease appeared to be much more significant in the division zone, the lowest part of internode corresponding to the intercalary meristem, compared to that in elongating zone (Figure 8F). In addition, the decrease was most obvious in IN2 compared to IN1, particularly of the double mutant (Figure 8F), consistent with the more severe shortening of IN2 in the mutants.

### BR levels were upregulated in seedlings and panicles but were downregulated in internodes in the mutants

We also analyzed the expression of several BR biosynthetic genes in panicles, IN1 and IN2. Surprisingly, these genes generally had increased expression in panicles, but had decreased expression in internodes, suggesting an intriguing possibility that BR levels could be oppositely regulated in different tissues (Supplemental Figure S13).

**Figure 9:**
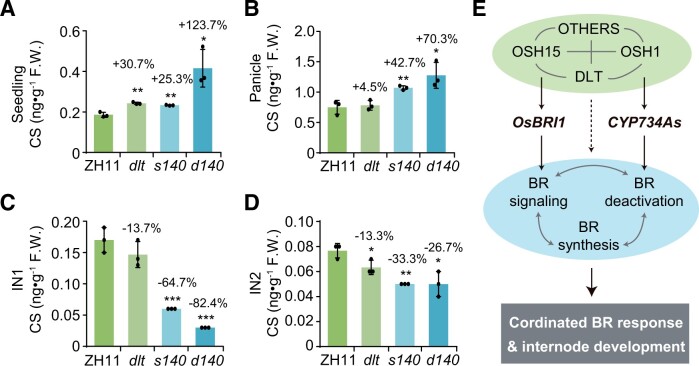
The DLT–OSH15 complex regulates BR contents in a tissue-specific manner. A–D, CS levels in ZH11, *dlt*, *s140*, and *d140* in different tissues, including 15-day-old seedlings (A), panicles (B), IN1 (C), and IN2 (D) at the heading stage. F.W., fresh weight. Data are shown as means ± sd (*n* = 3). Asterisks indicate significant difference compared with ZH11, with *P < 0.05, **P < 0.01, and ***P < 0.001 by Student’s two-sided *t* test. The increase (+) or decrease (−) percentages compared with ZH11 were shown on the columns. E, Proposed model integrating the findings of this study and the previous studies. DLT and OSH15 together with many additional components form a protein complex (upper oval), which regulates BR function by targeting *OsBRI1* and the BR catabolic genes *CYP734As* (Tsuda et al., 2011 as well as other BR-related genes (dashed arrow). BR signaling and BR metabolism also affect each other (double sided arrows in middle oval). The DLT–OSH15 complex confers switchable regulation on downstream genes to coordinate BR response and internode elongation.

Since BR metabolism and BR signaling regulate each other in a complex manner, it is difficult to determine how BR levels are ultimately affected in plants based on expression analysis. Therefore, we directly quantified the BR contents in various tissues, including seedlings, panicles, IN1, and IN2, in mutant and wild-type plants (Figure 9, A–D; Supplemental Table S1). Castasterone (CS) is one of the major active BRs whose contents could be measured in all of these tissues. In seedlings, both *dlt* and *s140* had significantly increased levels of CS compared to the wild-type (Figure 9A). Importantly, *d140* had a markedly increased level of CS (123.70% increase compared with the wild-type), which were much higher than that predicted by a simple additive model involving the two genes (30.70% increase in *dlt* and 25.31% in *s140*; Figure 9A). Therefore, although OSH15 alone has only a minor effect on CS level at the seedling stage, it appears to have a significant effect at the physiological level by interacting with DLT. This notion is also supported by the finding that *d76* plants are shorter than *dlt* plants, further suggesting that DLT and OSH15 regulate BR responses in a synergistic manner. We obtained similar results for CS levels in panicles, as the double mutant had much higher increases in CS contents (70.34% increase versus the wild-type) than the single mutants (4.45% increase in *dlt* and 42.37% in *s140*; Figure 9B).

In contrast, in both IN1 and IN2, the CS contents were strongly reduced in most of the mutants (Figure 9, C and D; Supplemental Table S1). This discovery demonstrated that DLT and OSH15 play different roles in regulating BR responses in different tissues. Strikingly, compared to the single mutants, the CS levels showed a synergistic decrease in IN1 of *d140* (Figure 9C), in contrast to the observation in seedlings, which showed a synergistic increase of CS contents (Figure 9A). In IN2, however, the CS levels in *d140* were similar to those in *s140*, although CS levels were also reduced in *dlt* (Figure 9D), suggesting that *OSH15* is epistatic over *DLT* in regulating BR biosynthesis in this specific tissue, which is consistent with the IN2 phenotypes of the mutants.

## Discussion

In this study, we demonstrated that DLT and OSH15 cooperatively interact with each other to regulate the expression of genes, including *OsBRI1*, to modulate BR responses and the development of different tissues including internodes (Figure 9E). Coincidently, it has been shown that OSH1 promotes the expression of BR catabolism genes in rice to suppress BR biosynthesis in the shoot apical meristem, and DLT also interacts with OSH1 to regulate cell division (Yang et al., 2018). The overexpression of *OSH15* led to severe defects in shoot development (Supplemental Figure S11), supporting the notion that OSH15, like OSH1 (Tsuda et al., 2011), is able to function in the shoot apical meristem. These results strongly suggest the intense functional association among these proteins in different tissues. In addition, DLT is thought to play a similar role to BZR1/BRI1-EMS-SUPPRESSOR1, which target BR biosynthetic genes to suppress BR production (Tong and Chu, 2009). Both DLT and OSH15 can interact with themselves as well as many other transcription factors such as OSH members, OFP members, SMOS1/RLA1, etc. (Hirano et al., 2017; Qiao et al., 2017; Xiao et al., 2017; Yang et al., 2018). Therefore, these components might form a large transcription factor complex, with switchable relationships among components (Figure 9E), to dynamically regulate various BR responses according to internal growth demands or external environmental conditions (Tong and Chu, 2018).

In a well-known feedback mechanism, defects in BR signaling usually result in the elevated BR synthesis due to decreased expression of BR biosynthetic genes and/or increased expression of BR catabolic genes (Figure 9E). However, in all the single and double mutants, while both seedlings and panicles contained increased average BR contents compared to the wild-type, the internodes unexpectedly contained reduced BR contents. Since long-distance BR transport within the plant is not well documented (Symons and Reid, 2004; Symons et al., 2008), it is unlikely that this opposite pattern of BR accumulation in panicles and internodes (two adjacent plant parts) is caused by BR transport. Hence, our findings strongly suggest that the DLT–OSH15 complex plays variable roles in regulating BR responses (BR metabolism). This result is consistent with the tissue phenotypes of the mutants, as both *osh15* and *dlt* had increased grain size but shortened internodes.

The differential regulation of BR levels in different tissues by the DLT–OSH15 complex might be achieved via opposite regulatory effects on the same genes. A more feasible possibility is that the DLT–OSH15 complex simultaneously targets different genes affecting BR homeostasis in distinct ways (Supplemental Figure S12) and has different regulatory biases in different tissues, which could be at least partially attributed to the specific expression patterns of *DLT* and *OSH15*, since *DLT* is expressed at relatively low levels in IN2 whereas *OSH15* is expressed at significantly high levels in IN2 (Figure 4, B–D). The biased regulation could also result from a compensation effect among OSH members, a concept that has been proposed (Sato et al., 1999). Indeed, we found that at least three close homologous genes of *OSH15* have specifically enhanced expression in IN1 but not IN2 (Supplemental Figure S14A), which may compensate for the defects of *osh15* in IN1. In addition, all the three proteins can bind the *OsBRI1* promoter region, as revealed by yeast one-hybrid analysis (Supplemental Figure S14B) and, like OSH15, can enhance the promoter activity as revealed by luciferase reporter analyses (Supplemental Figure S14C). Furthermore, the differential regulation could also be achieved by integrating or recruiting additional components of the complex, as has been discussed above.

We hypothesize that DLT and OSH15 function in a synergistic manner, at least in some tissues. The *d76* mutant showed strongly reduced seedling height compared to *dlt*, whereas *s76* was only slightly dwarfed compared to the wild-type. This hypothesis is also supported by the transcriptome analysis: Although *s140* showed few mutant phenotypes at the seedling stage, a high proportion of genes were commonly upregulated in *dlt*, *s140*, and *d140*, with the lowest amplitude of changes in *s140*, followed by *dlt* and *d140*. This hypothesis is further supported by the BR quantification analysis: According to multiple comparison analysis, little increase in BR content was detected in *dlt* and *s140*, but *d140* contained significantly higher BR contents than the wild-type. Notably, the balance between BR signaling and BR biosynthesis is dynamically regulated in the mutants, resulting in unpredictable phenotypes. For example, at the seedling stage, *s140* contains increased BR contents but has decreased BR signaling, resulting in slightly increased leaf angle and plant height. This observation is reminiscent of the phenotypes of *bzr1-D* in *Arabidopsis thaliana*, a gain-of-function mutant of BZR1; this critical transcription factor plays a positive role in BR signaling, but the mutant shows reduced stature due to suppressed BR biosynthesis (Wang et al., 2002).

In maize (*Zea mays*), BLH12 and BLH14, two BELL1-like homeobox proteins that interact with KNOTTED1, control precocious internode differentiation (Tsuda et al., 2017). Our findings for *s140* suggest that OSH15 plays a similar role in rice, suggesting that BRs are involved in this process. OSH15 is thought to interact with other BELL-like proteins to suppress lignin biosynthesis to control seed shattering in rice (Yoon et al., 2017), an important agronomic trait that has undergone domestication. Interestingly, BRs also regulate lignin levels, a process associated with cell wall remodeling (Rao and Dixon, 2017). Our discovery that OSH15 interacts with DLT to regulate BR responses suggests a novel mechanism whereby BRs regulate lignin biosynthesis or seed shattering.

*DLT* and *OSH15* are the responsible genes for the multiple allelic dwarf mutants known as *dlt*/*d62/grain size6/smos2* and *d6/osh15*, respectively, thus functioning as critical regulators of plant height (Sato et al., 1999; Tong et al., 2009; Li et al., 2010; Sun et al., 2013; Hirano et al., 2017; Yoon et al., 2017). The discovery that DLT and OSH15 function as a complex to modulate the differential elongation of rice internodes suggests that the DLT–OSH15 interaction plays a key role in mediating this important yet complex process. Moreover, by performing extensive genetic, transcriptome, and hormonal analyses, we revealed how the DLT–OSH15 complex regulates this process at the molecular and physiological levels. We also uncovered the different relationships between DLT and OSH15 in different tissues, including different internodes, and the process underpinning the differential elongation of internodes in coordination with developmental requirements. We believe that the variable relationship between two interacting proteins uncovered in this study represents a fundamental mechanism that plants adopt to coordinate growth and development. Thus, this study greatly increases our understanding of the molecular basis of key developmental events.

In the field, even within a single plant, internodes usually elongate at different times in different tillers under different growth conditions. Also, different rice germplasms show distinct tiller elongation patterns. It is not easy to collect the desired internode samples for comparative analysis due to the complexity of internode development. Nevertheless, coordinated elongation of internodes is crucial for the development of other organs, including leaves and panicles. Therefore, the mechanism uncovered in this study moves an important step toward understanding this process. Several studies have suggested that GA plays an important role in controlling the length of the uppermost internode (Luo et al., 2006; Gao et al., 2016). In contrast, BR appears to play a significant role in the lower internodes, especially IN2. Given the known crosstalk between BR and GA (Li et al., 2012; Tong et al., 2014; Unterholzner et al., 2015), it is possible that the two hormones interact to coordinate the development of these two adjacent parts of the culm, a concept currently under investigation in our laboratory. Finally, internode elongation is highly intertwined with grain development. Given the predominant roles of BRs in regulating both grain size and the differential elongation of internodes, it will be highly valuable to tease apart the molecular mechanisms underlying these two important agronomic traits. Such information would facilitate crop improvement via the specific manipulation of BR function in certain tissues, as proposed recently (Tong and Chu, 2018). Notably, both *osh15* alleles show semidwarf statures due to shortening of the lower internodes, with minor effects on leaf morphology, but they exhibit enlarged grains and increased tiller number, highlighting the specific regulation of BR responses and representing valuable resources for crop improvement.

## Materials and methods

### Plant materials and mutagenesis

The *O.sativa japonica* cultivar ZH11 was used as the wild-type. Plants were grown in the field under natural conditions for agronomic trait analysis or in a growth chamber in half-strength Murashige and Skoog nutrient solution for seedling analysis. The diurnal cycle was set to 14-h light (30°C)/10-h dark (28°C) with light intensity of ∼150 µmol m^−2^s^−1^ (white light) and 60% relative humidity. For mutagenesis, dry *dlt* seeds (∼0.75 kg) were incubated in 1-mM sodium azide (pH 3.0, 1 M KH_2_PO_4_ and 1 M H_3_PO_4_) for 6 h and thoroughly rinsed with tap water. The treated seeds were germinated as usual and planted in the field. The seeds were harvested from each plant and sown separately in the field (16 plants per line) for phenotyping and mutant screening.

### MutMap cloning

The sequencing data were processed with Trimmomatic (version 0.36; Bolger et al., 2014) to remove adaptors and low-quality reads. The resulting clean reads were mapped to the Nipponbare rice reference genome (version 7.0; Kawahara et al., 2013) with bwa (version 0.7.13-r1126; Li and Durbin, 2009), followed by gap filling using GATK (version 3.5; DePristo et al., 2011). The SNPs across the genome were called using SAMtools (version 0.1.19; Li et al., 2009). The SNPs were further filtered by removing shared SNPs with wild-type ZH11 and the other mutant pool. For the remaining SNPs, the SNP indexes were calculated as the allele frequency of the specific mutation in the mutant pool using high-quality data (mapping quality ≥30 and base quality ≥20).

### Vector construction

See Supplemental Table S3 for the primer sequences and empty vectors used for vector construction. The full-length cDNAs encoding OSH15, DLT, and OSH15 homologs were used to construct various vectors for different experiments as well described below, including yeast two-hybrid, subcellular localization, split-luciferase complementation, Co-IP, yeast one-hybrid, and luciferase reporter assays. The cDNAs were amplified using the corresponding primers and introduced in the target empty vectors by in-fusion cloning strategy. Transgenic plants were produced by *Agrobacterium tumefaciens*-mediated transformation.

### RT-qPCR

Total RNA was isolated from various tissues using TRIzol reagent (Invitrogen, USA), and cDNAs were prepared using a reverse transcription kit (ReverTra Ace qPCR RT Master Mix with gDNA Remover) following the manufacturer’s instructions (Toyobo, Japan). RT-qPCR was performed on a LightCycler96 machine (Roche, USA) using SYBR Green Master Mix (Roche, USA). The relative expression levels of the genes were calculated based on 2^−ΔΔCт^ values normalized to *UBIQUITIN* mRNA levels. All primer sequences are listed in Supplemental Table S4.

### GUS staining

Construction of *DLTp:GUS* has been reported previously (Tong et al., 2009). *OSH15p:GUS* was generated by the same way. Two-kb promoter region of *OSH15* was amplified with the primer sequences listed in Supplemental Table S3. The vectors were introduced into ZH11 by *A.tumefaciens*-mediated transformation. The tissues or hand-cut sections of the transgenic plants were incubated in commercial GUS staining solution at 37°C overnight, and distained in 75% ethanol. Images were taken directly or under the stereomicroscope (SZX16, Olympus).

### BR sensitivity test

For the lamina inclination test, ZH11, *dlt*, *s140*, and *d140* were grown for 3 days following 2 days of germination at 30°C. Ethanol (1 µL) containing 0, 10, 100, or 1,000 ng BL (Wako, Japan) was added to the lamina tip (Tong and Chu, 2017). After additional 3-day growth, the plants were photographed and the angles between the lamina and leaf sheath were measured using ImageJ software. For other BR sensitivity assays, seeds were germinated and grown on 1% agar medium supplemented with various concentrations of BL for 7 days at 30°C, then coleoptile length and root length were measured as reported previously (Tong and Chu, 2017).

### Protein subcellular localization

For subcellular localization analysis, rice protoplasts were prepared and transfected with plasmids as described previously (Bart et al., 2006). Fluorescence was observed under a confocal laser scanning microscope (Zeiss LSM 780). At least 10 cells showing consistent localization pattern were observed.

### Yeast two-hybrid, split-luciferase complementation, and Co-IP

See Supplemental Table S3 for the vector information and primers using for the constructions. Yeast two-hybrid assays were performed using the Golden Yeast System following the manufacturer’s instructions (Takara). Within 1 week of cultivation, yeast cells growing on SD/His–Ade–Leu–Trp– plates were counted as positive clones. Split-luciferase complementation assays were performed in *N.benthamiana* leaves as described (Chen et al., 2008). Chemiluminescence was photographed using an imaging system equipped with a cold CCD (NightSHADE LB985).

For Co-IP, different combinations of constructs were cotransfected into *N. benthamiana* leaves via *Agrobacterium*-mediated infiltration. After 2 days, total proteins were extracted from the infiltrated leaves in IP buffer [50-mM 2-amino-2-(hydroxymethyl)-1,3-propane diol (Tris)–HCl, pH 7.5, 150-mM NaCl, 0.5-mM EDTA, 0.2% Nonidet P-40, 0.6-mM phenylmethanesulfonyl fluoride (PMSF), and 1× Complete Protease Inhibitor Cocktail] and incubated with anti-GFP magnetic beads (Sigma, USA) for 3 h at 4°C in a top-to-end rotator. After incubation, the magnetic beads were washed three times with ice-cold washing buffer (50-mM Tris–HCl, pH 7.5, 150-mM NaCl, 0.6-mM PMSF, and 1× Complete Protease Inhibitor Cocktail) and eluted by boiling in sodium dodecyl sulfate sample buffer. The samples were separated by sodium dodecyl sulfate–polyacrylamide gel electrophoresis, detected by immunoblotting using anti-FLAG (M185-7, 1:3,000 dilution, MBL, USA) or anti-GFP (598-7, 1:3,000 dilution, MBL, USA) antibodies.

### Transcriptome sequencing

Various tissues, including seedlings, panicles, IN1 and IN2, of ZH11, *dlt*, *s140*, and *d140* were sampled for transcriptome analyses. For seedlings, aerial parts of 15-day-old seedlings cultured in growth chamber were sampled. For other tissues, panicles, IN1, and IN2 of field-grown plants at heading stage were sampled. For each sample, at least six individual plants were collected for RNA isolation. The sequencing libraries were constructed using a NEB Next Ultra RNA Library Prep Kit (NEB, USA) following the manufacturer’s instructions. Three replicates were prepared for the panicle, IN1, and IN2 samples, and one for seedlings. RNA sequencing (RNA-seq) and data analyses were performed on an Illumina HiSeq 6000 instrument using an established bioinformatics pipeline developed by the Annoroad Gene Technology Corporation (Beijing, China). Briefly, raw reads from the samples were first processed to generate clean reads. Bowtie2 (v2.2.3) was used for building the genome index, and the clean data was aligned to reference genome using HISAT2 (v2.1.0; Kim et al., 2015). Reads count for each gene in each sample was counted by HTSeq (v0.6.0), and fragments per kilobase million mapped reads was calculated to estimate the expression level of genes in each sample (Wagner et al., 2012). DESeq2 was used to estimate the expression level of each gene in per sample by the linear regression, then the *P*-value was calculated with Wald’s test and corrected by the BH method (Wang et al., 2010). Genes with *P* ≤ 0.05 and log2_ratio ≥1 were identified as DEGs. The RNA-seq data were deposited in Beijing Institute of Genomic Data Center (http://bigd.big.ac.cu).

### Cluster, GO, and TO analyses

Log2FC values of the overlapping downregulated DEGs among *dlt*, *s140*, and *d140* were used for cluster analyses. The heatmap plot and hierarchical clustering were generated by the R package “pheatmap” with default parameters for clustering method (complete) and clustering distance (Euclidean). GO annotations are manually curated and collected from three databases (update until July 16, 2019): Oryzabase (https://shigen.nig.ac.jp/), RAPDB (https://rapdb.dna.affrc.go.jp/), and ricedata (http://www.ricedata.cn/). TO annotations are also manually curated and are collected from the Oryzabase and ricedata websites. Each DEG list was compared with a background gene list which includes only genes that were detectable in the RNA-seq data set (at least two read counts). The enrichment or depletion of each ontology term was tested using the Fisher’s exact test. To correct for multiple tests, we performed 1,000 permutations and ask what is the probability in the permutation that we observe more or equal extreme cases than that observed for the data (the corrected *P*-value).

### Yeast one-hybrid assay

See Supplemental Table S3 for the vector information and primers using for the constructions. The full-length cDNAs encoding OSH15, DLT, and OSH15 homologs were cloned into the pB42AD vector. Approximately 2.5 kb and various truncated versions of *OsBRI1* promoter were cloned into the Placzi2u vector. Combinations of different constructs were transformed into the yeast strain EGY48. After growing on selective dropout media for 3 days at 30°C, the transformants were transferred onto 5-bromo-4-chloro-3-indolyl-β-d-galactopyranoside plates for further development. Combinations containing the empty pB42AD were used as negative controls.

### Luciferase reporter assay

See Supplemental Table S3 for the vector information and primers using for the construction. The effector vectors and reporter vectors were transformed into *Agrobacterium* strain AGL1 respectively, and then introduced into *N. benthamiana* leaves. After 2-day growth, LUC activity was quantified with a Dual-Luciferase Assay Kit (E1910, Promega) following the manufacturer’s recommendations.

### ChIP-seq and ChIP-qPCR

ChIP assay was performed as described previously with minor modifications (Duan et al., 2019; Yin et al., 2020) . For ChIP-seq, transgenic calli (∼4 g) with the vector expressing OSH15–FLAG or empty FLAG vector were prepared for isolating protein–DNA complex using anti-FLAG antibodies (F1804, 1:3,000 dilution, Sigma-Aldrich). Isolation of genomic DNA, sequencing, and further data analysis were performed as described previously. Vectors expressing OSH15–FLAG and *OsBRI1* gene containing the 2.5-kb promoter were introduced into *N. benthamiana* leaves for ChIP-qPCR. See Supplemental Table S3 for the vector information and primers using for the construction. Primers for qPCR are listed in Supplemental Table S4. The ChIP-seq data were deposited in Beijing Institute of Genomic Data Center (http://bigd.big.ac.cu).

### BR quantification

Seedling, panicle, IN1, and IN2 samples (1.5 g) were collected same with those in RNA-seq analyses from ZH11, *dlt*, *s140*, and *d140* for hormonal measurements following previous descriptions (Ding et al., 2013). BL, Castasterone (CS), and 28-homoCS were quantified, and only CS was successfully detected in all the tissues.

### Statistical analyses

Student’s two-tailed *t* test was used for significant difference analysis between two samples. For comparison of the DEG regulation, paired *t* test with unequal variance hypothesis was used to generate *P*-values. One-way ANOVA analyses followed with Tukey’s test (*P* < 0.05) were used for pairwise multiple comparisons. All the analyses were performed using SPSS software. Data for all statistical analyses are shown in Supplemental Data Set S6.

### Accession numbers

Sequences of genes involved in this study can be found in Rice Genome Annotation Project (http://rice.plantbiology.msu.edu/) under the accession numbers LOC_Os07g03770 (OSH15), LOC_Os06g03710 (DLT), LOC_Os01g52050 (OsBRI1), LOC_Os01g10040 (D2), LOC_Os04g39430 (D11), LOC_Os03g40540 (BRD1), LOC_Os03g51690 (OSH1), LOC_Os03g51710 (OSH3), and LOC_Os03g56110 (OSH43). Sequencing data of RNA-seq (Bioproject PRJCA001931) can be found in Beijing Institute of Genomics Data Center (http://bigd.big.ac.cn) under the accession number CRA002126. Sequencing data of ChIP-seq (Bioproject PRJCA008386) can be found in Beijing Institute of Genomics Data Center (http://bigd.big.ac.cn) under the accession number CRA006147.

## Supplemental data

The following materials are available in the online version of this article.

**Supplemental Figure S1.** The SNP index plot for *s76* on the 12 rice chromosomes.

**Supplemental Figure S2.** The SNP index plot for *s140* on the 12 rice chromosomes.

**Supplemental Figure S3.** Colocalization analyses of DLT and OSH15.

**Supplemental Figure S4.** Interaction between OSH15 and itself.

**Supplemental Figure S5.** Comparison of the cross sections of IN1 in different plants.

**Supplemental Figure S6.** DLT and OSH15 exhibit differential dominance in regulating gene expression in different tissues.

**Supplemental Figure S7.** Expression analysis of selected DEGs in different tissues of different plants.

**Supplemental Figure S8.** Shared GO terms enriched using downregulated DEGs in IN2 of different mutants.

**Supplemental Figure S9.** Shared TO terms enriched using downregulated DEGs in IN2 of different mutants.

**Supplemental Figure S10.** Differentially expressed BR-related genes in different tissues of different plants.

**Supplemental Figure S11.** Phenotypes of *OSH15*-overexpression plants.

**Supplemental Figure S12.** Visualization of the binding peaks in ChIP-seq.

**Supplemental Figure S13.** Expression of BR biosynthetic genes in different tissues.

**Supplemental Figure S14.** OSH homologs might compensate for OSH15 defection in IN1.

**Supplemental Table S1.** Hormone quantification data.

**Supplemental Table S2.** Statistical analysis of seedling height, IN1 length, IN2 length, and tiller number in different plants.

**Supplemental Table S3.** Information for vector construction.

**Supplemental Table S4.** Primers used for RT–qPCR.

**Supplemental Data Set S1.** DEG lists in seedlings of the three mutants.

**Supplemental Data Set S2.** DEG lists in IN1 of the three mutants.

**Supplemental Data Set S3.** DEG lists in IN2 of the three mutants.

**Supplemental Data Set S4.** Lists of significantly enriched GO terms in IN1 and IN2 of different mutants.

**Supplemental Data Set S5.** Lists of significantly enriched TO terms in IN1 and IN2 of different mutants.

**Supplemental Data Set S6.** Summary of statistical tests.

## Funding

This work was supported by the National Natural Science Foundation (nos. 31901534, 31900177, 31871587, 31722037, U21A20208), Hainan Yazhou Bay Seed Lab (B21HJ0215), Central Public-interest Scientific Institution Basal Research Fund (nos. S2022ZD02, Y2020XK16, S2021ZD01), and China Postdoctoral Science Foundation (2018M641554).

*Conflict of interest statement*. None declared.

## Supplementary Material

koac196_Supplementary_Data

## Notes

H.T. conceived and designed the study and carried out the initial identification of the mutants. H.W. developed the MutMap pipeline and performed bioinformatics analyses. F.Z. assisted the analyses. M.N. conducted most of the other experiments. W.Y., W.M., Y.X., D.L., X.Z., N.D., and J.L. provided assistance. H.T., M.N., and H.W. analyzed the data and wrote the manuscript with input from all the other authors. H.T. and C.C. supervised the study.

The author responsible for distribution of materials integral to the findings presented in this article in accordance with the policy described in the Instructions for Authors (https://academic.oup.com/plcell) is: Hongning Tong (tonghongning@caas.cn).
