# Supplementary material for: Rice DWARF AND LOW-TILLERING and the homeodomain protein OSH15 interact to regulate internode elongation via orchestrating brassinosteroid signaling and metabolism
Source: Plant Cell. 2022 Jul 5;34(10):3754–72. doi: 10.1093/plcell/koac196 (PMC9516196; doi:10.1093/plcell/koac196)
Supplement: koac196_Supplementary_Data [file koac196_supplementary_data.zip › koac196_Supplementary_Data/tpc.21.01109Supplemental Figures and Tables.pdf]

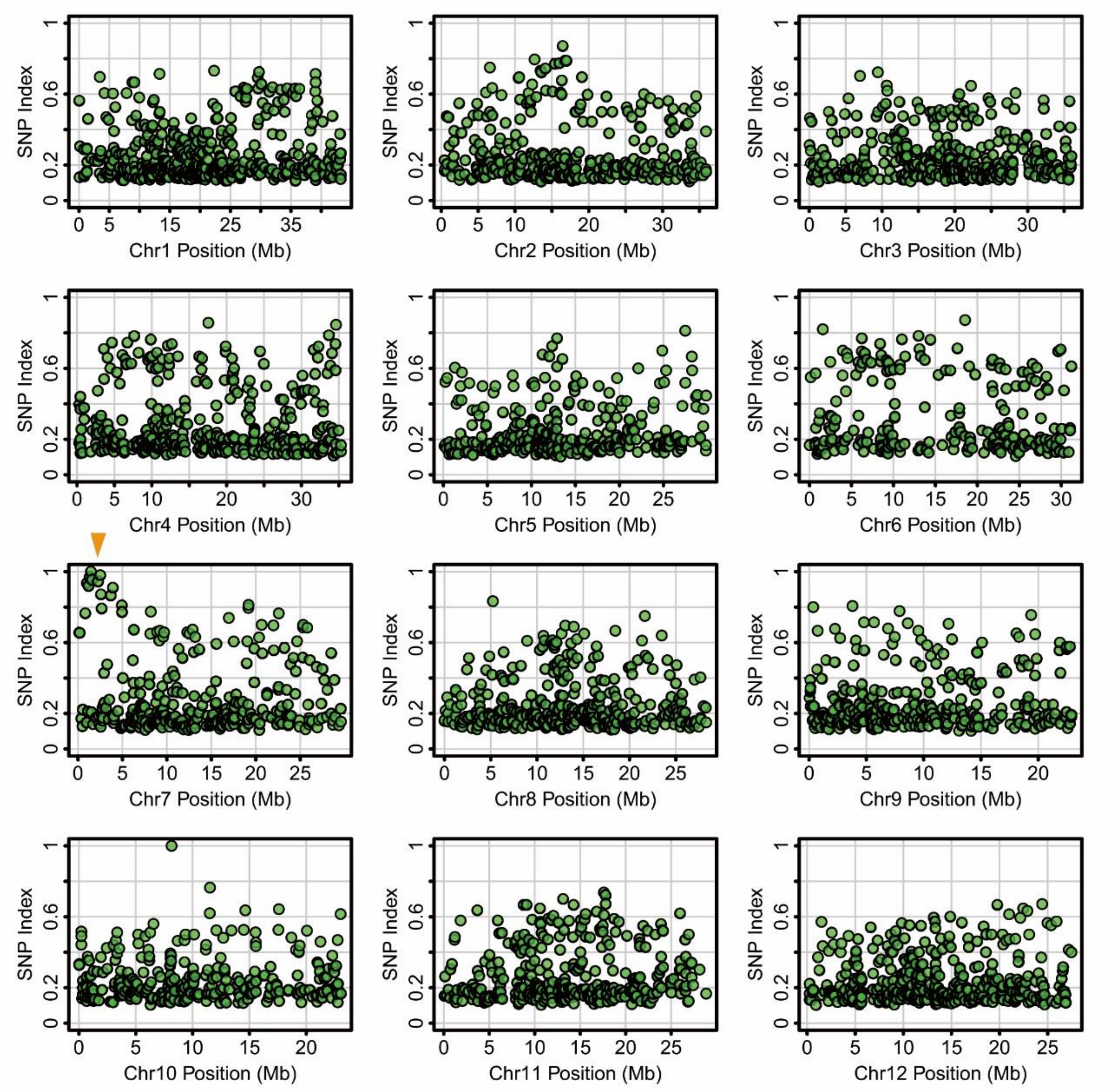

Supplemental Figure S1. The SNP index plot for *s76* on the 12 rice chromosomes.
(Supports Figure 2)
Each dot on the plot represents a mutation and its corresponding allele frequency (SNP index) in the mutant pool. The expected pattern of the peak region for candidate mutation is only observed on chromosome 7, where SNP index peaks at candidate causal mutation and decreasing for more distant mutations due to recombination.

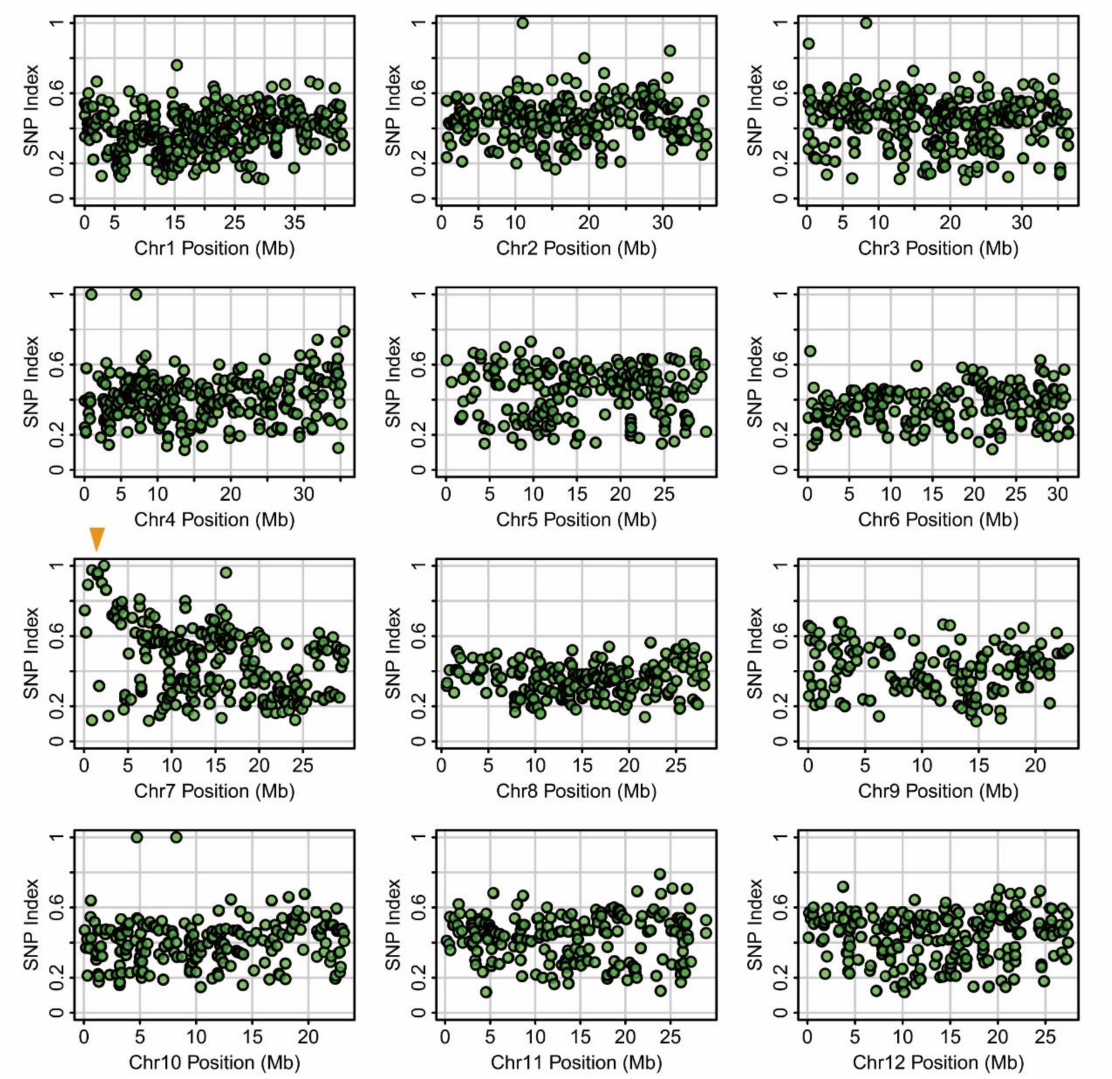

Supplemental Figure S2. The SNP index plot for *s140* on the 12 rice chromosomes. (Supports Figure 2)

Each dot on the plot represents a mutation and its corresponding allele frequency (SNP index) in the mutant pool. The expected pattern of the peak region for candidate mutation is only observed on chromosome 7, where SNP index peaks at candidate causal mutation and decreasing for more distant mutations due to recombination.

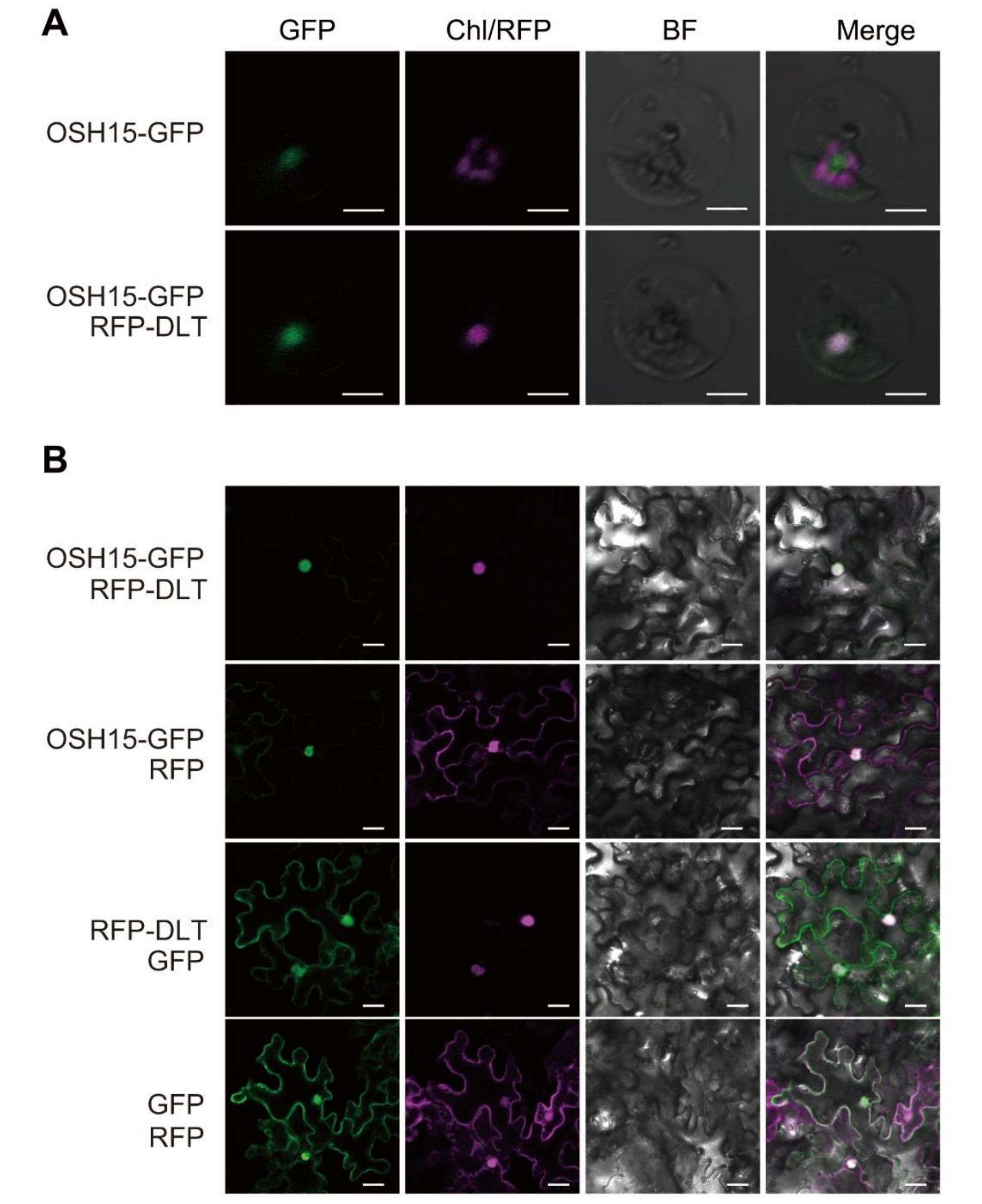

Supplemental Figure S3. Colocalization analyses of DLT and OSH15. (Supports Figure 4)
(A) OSH15-GFP and RFP-DLT were colocalized in nucleus in rice protoplast. BF, bright field; Chl, chlorophyll. Bar = 10 mm.
(B) OSH15-GFP and RFP-DLT were colocalized in nucleus in *N. benthamiana* leaves. BF, bright field; Chl, chlorophyll. Bar = 20 mm.

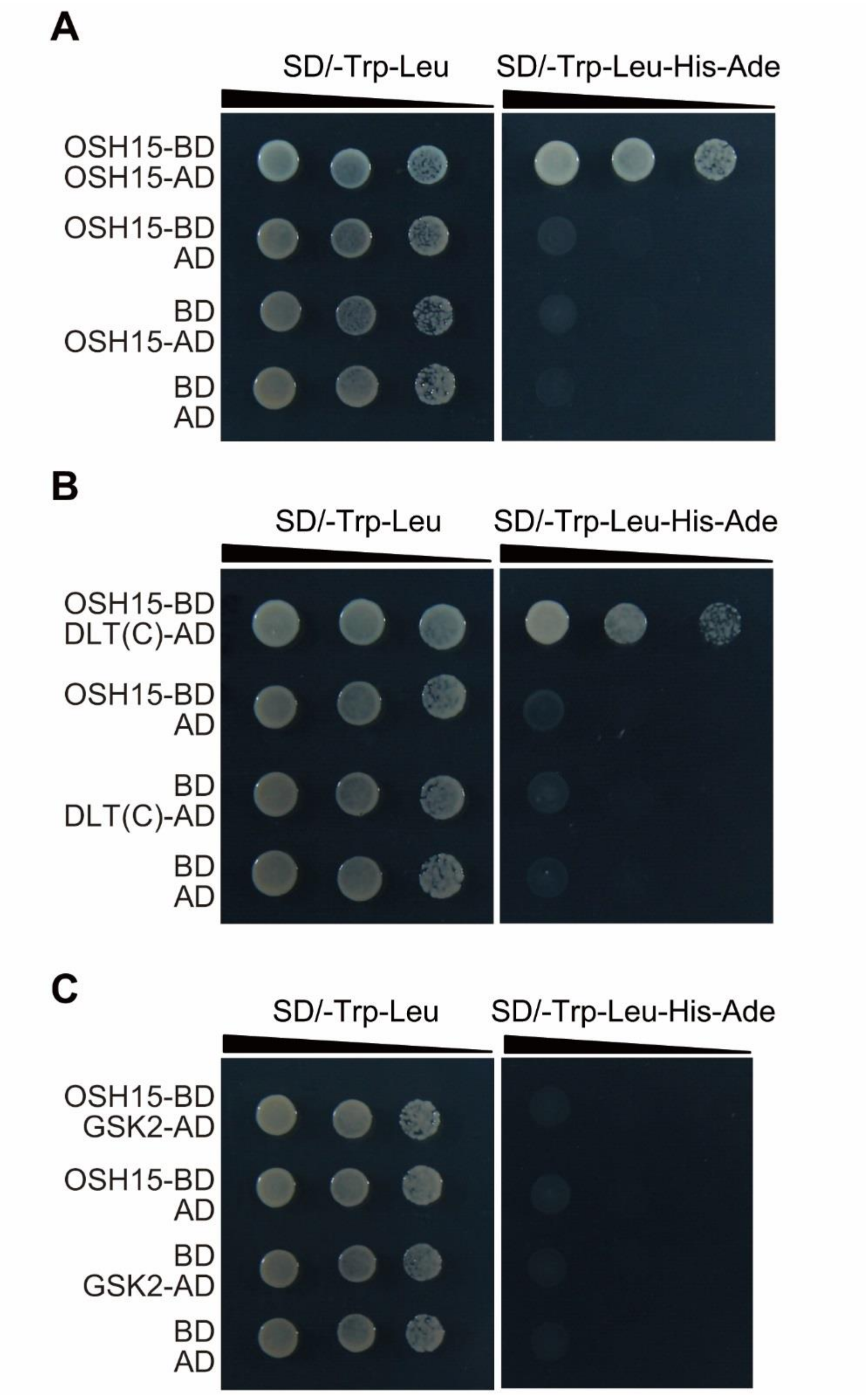

Supplemental Figure S4. Interaction between OSH15 and itself. (Supports Figure 4)

(A) Interaction between OSH15 and itself in yeast two-hybrid assay.

(B) Interaction between OSH15 and the C-terminal of DLT in yeast two-hybrid assay.

(C) OSH15 did not interact with GSK2 in yeast two-hybrid assay.

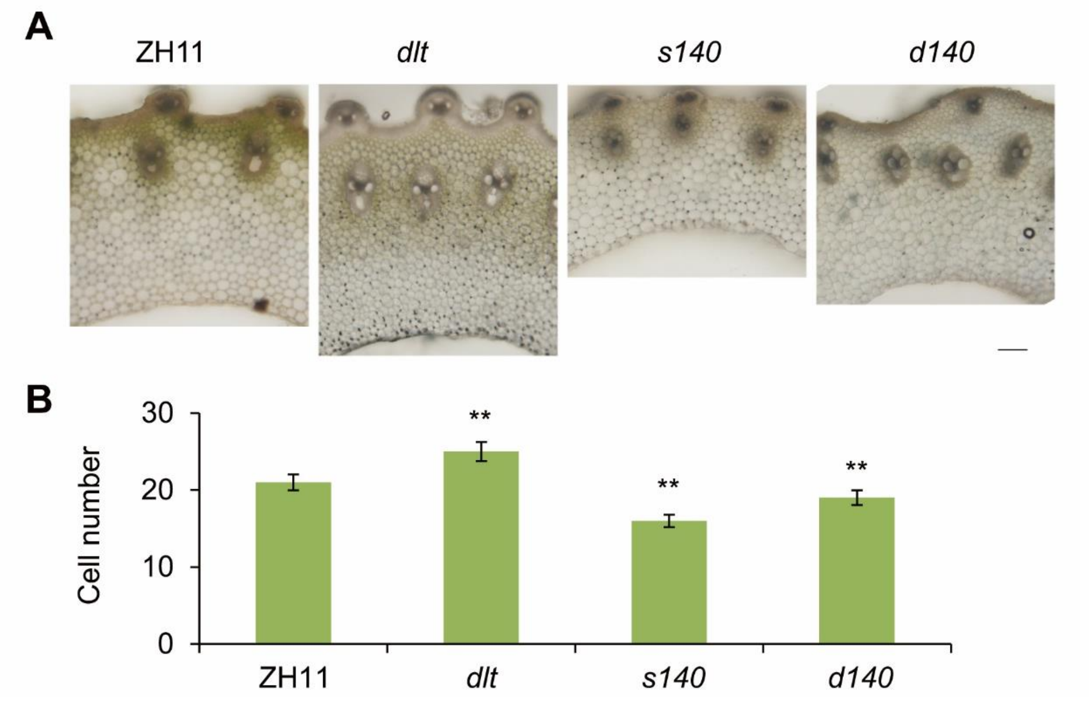

Supplemental Figure S5. Comparison of the cross sections of IN1 in different plants. (Supports Figure 6)

(A) Cross sections of IN1 of ZH11, *dlt*, *s140*, and *d140* after 95-d growth. Bar = 100 mm.

(B) Quantification of cell numbers of IN1 in (A). Data were shown as means ± SD (*n* = 12). Asterisks indicate significant difference compared with ZH11, with **P < 0.01 by Student's two-sided *t*-test.

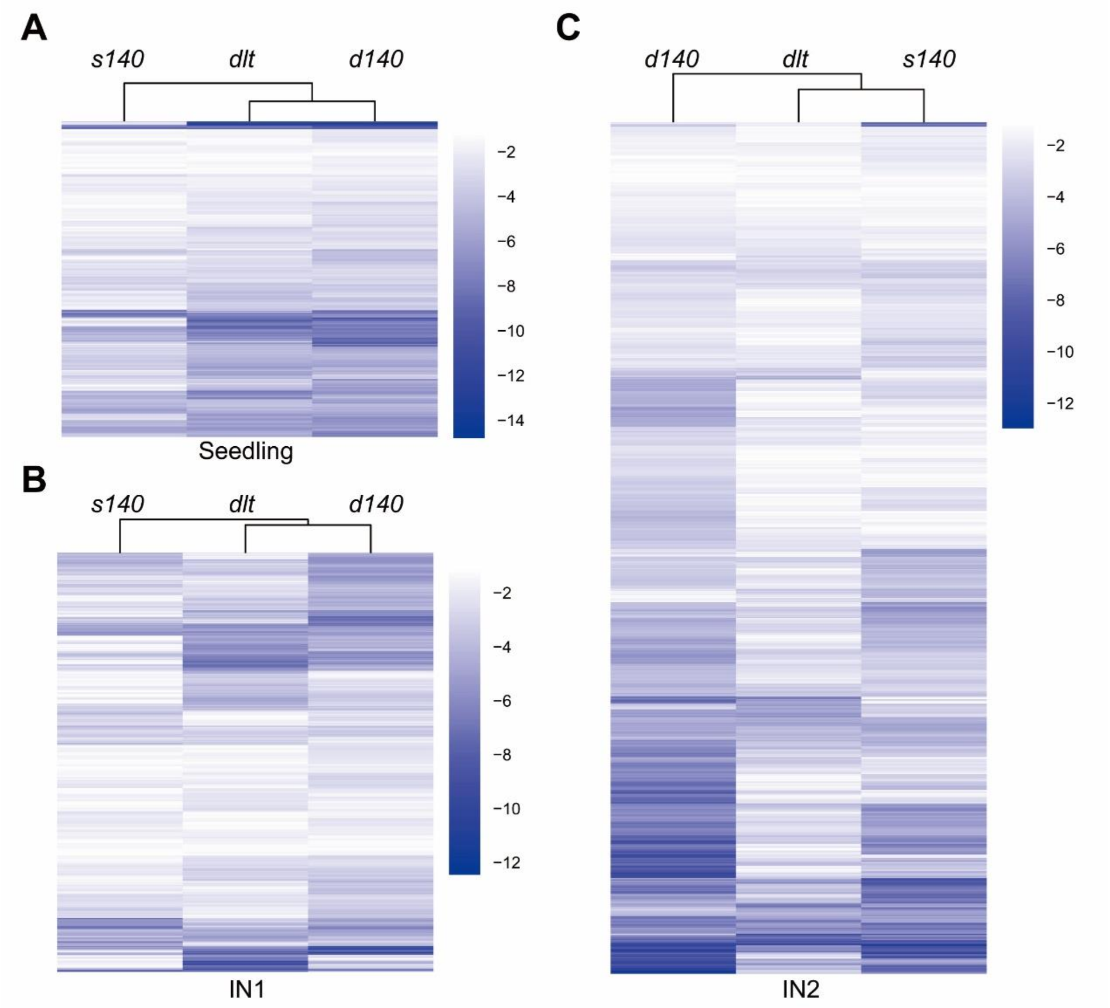

Supplemental Figure S6. DLT and OSH15 exhibit differential dominance in regulating gene expression in different tissues. (Supports Figure 7)

(A–C) Heatmaps showing the expression patterns of the co-downregulated DEGs in seedlings (A), IN1 (B), and IN2 (C) in different mutants. Colors indicate the logarithmic values of the fold changes (Log2FC) according to the color scale on the right. Clustering was generated by complete linkage method, with similarity metric assessed by Euclidean distance.

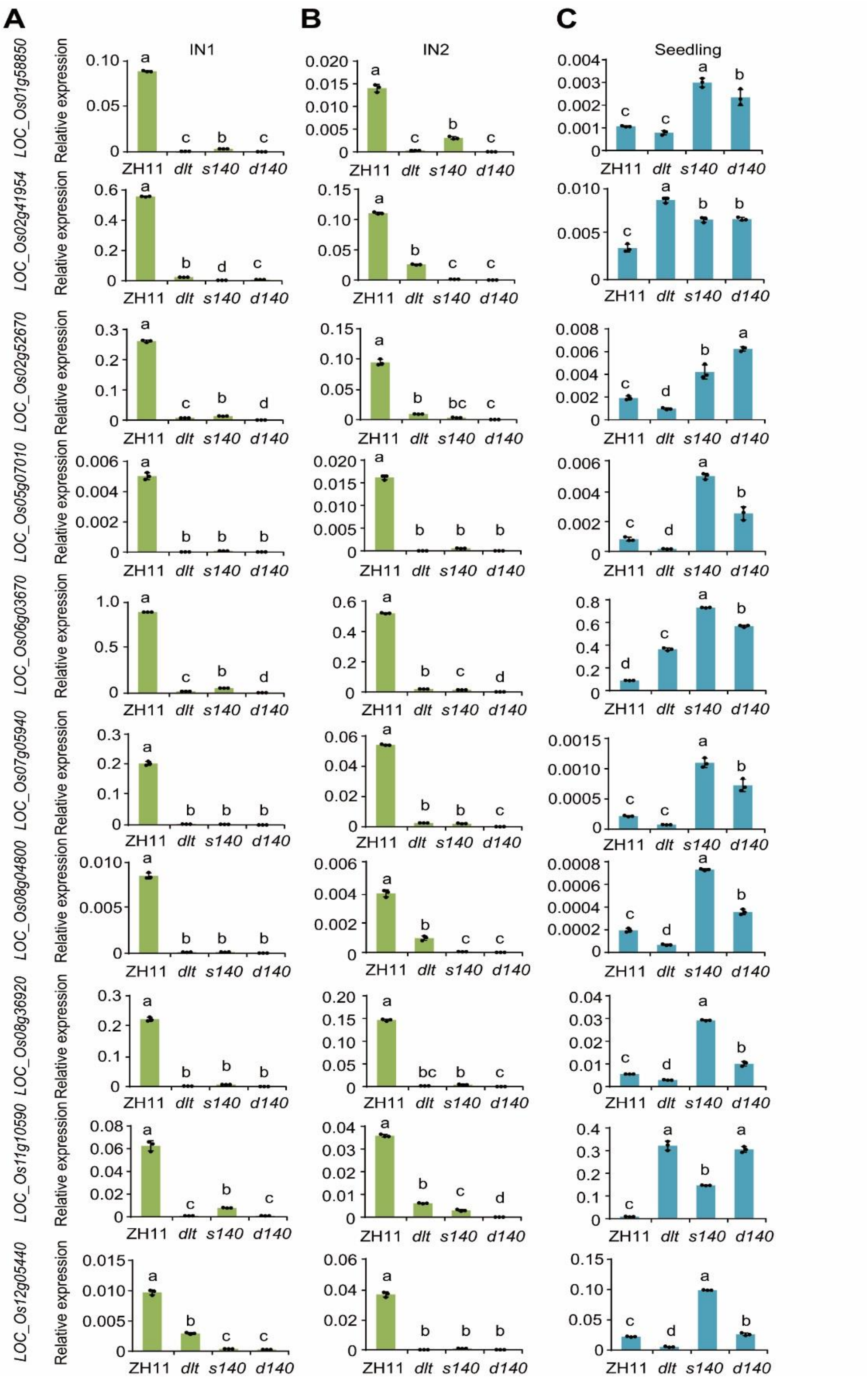

Supplemental Figure S7. Expression analysis of selected DEGs in different tissues of different plants. (Supports Figure 7)

IN1 (A), IN2 (B) and 15-d-old seedling (C) of ZH11, *dlt*, *s140* and *d140* were used for RT-quantitative PCR analysis. Data are shown as means ± SD (n = 3). Different letters on the histograms indicate statistically significant differences at $P < 0.05$ by pairwise multiple comparison followed with Tukey's test.

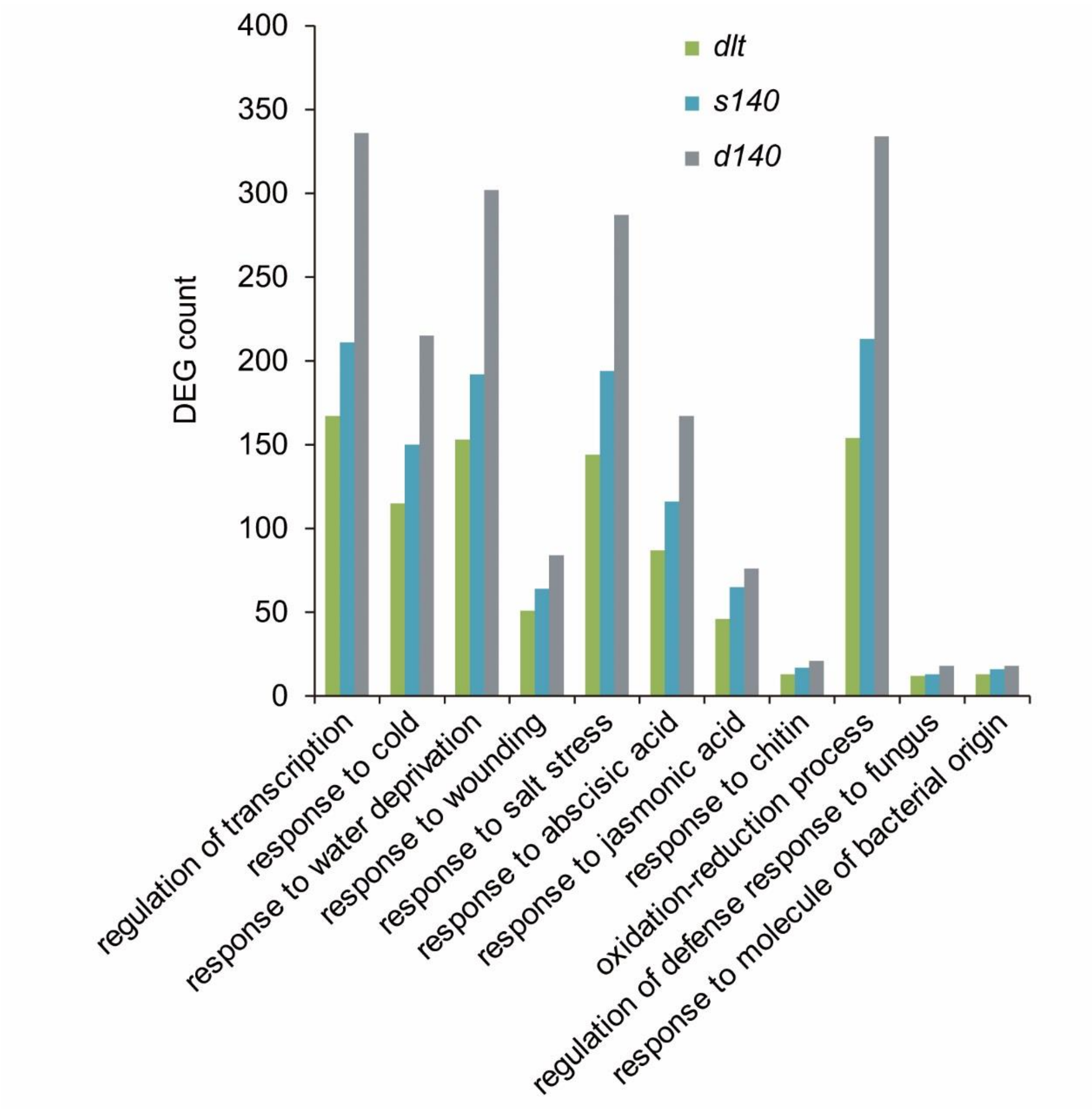

Supplemental Figure S8. Shared GO terms enriched using downregulated DEGs in IN2 of different mutants. (Supports Figure 7)

Significant GO terms (corrected P value < 0.05) enriched in all the three mutants are shown. See Supplemental Dataset 4 for more details.

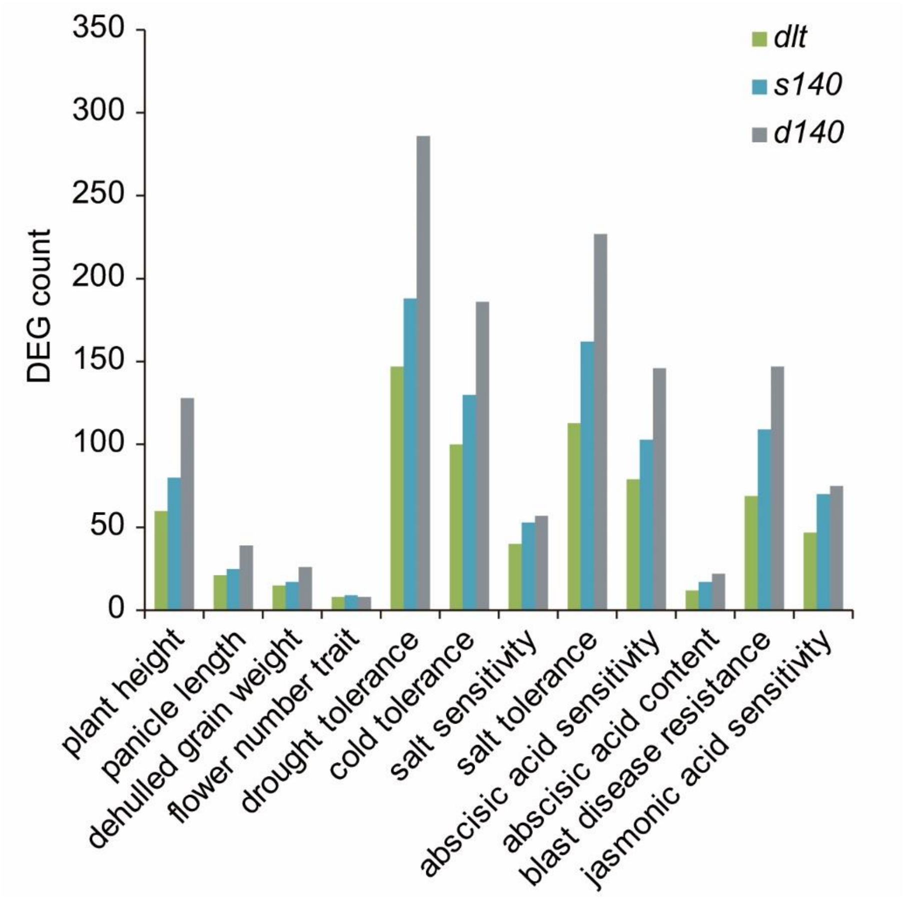

Supplemental Figure S9. Shared TO terms enriched using downregulated DEGs in IN2 of different mutants. (Supports Figure 7)

Significant TO terms (corrected P value < 0.05) enriched in all the three mutants are shown. See Supplemental Dataset S5 for more details.

	Tissue		**Seedling**			**IN1**			**IN2**		
Pathway	Name	LOC	*dlt*	*s140*	*d140*	*dlt*	*s140*	*d140*	*dlt*	*s140*	*d140*
BR synthesis	*D2*	*LOC_Os01g10040*					1.16	2.41	1.18		
	D11	*LOC_Os04g39430*				1.01		1.11			1.46
	DWARF4	*LOC_Os03g12660*				-2.85	-1.34	-2.50	-1.12		-4.17
	BRD1	*LOC_Os03g40540*		1.35						1.87	
	BRD2	*LOC_Os10g25780*				-3.73		-1.72			-2.03
BR catabolism	*CYP734A2*	*LOC_Os02g11020*			2.02						4.24
	CYP734A4	*LOC_Os06g39880*				-2.53		-2.70	-2.71	-1.49	-3.73
	CYP734A6	*LOC_Os01g29150*					1.52			2.59	
BR signaling	*BRI1*	*LOC_Os01g52050*									-1.06
	BRL1	*LOC_Os09g12240*							1.19	1.59	1.05
	BRL2	*LOC_Os10g02500*						-1.18			-1.33
	BRL3	*LOC_Os08g25380*				2.42	1.61	1.79			
	BAK1	*LOC_Os08g07760*				-1.86					
	BAK2	*LOC_Os03g61010*	-4.60	-1.64	-5.29	-3.49				1.23	-2.66
	SERK3	*LOC_Os06g12120*				-1.30	-2.03	-1.87	-2.48	-3.49	-2.37
	SERK4	*LOC_Os02g18320*				1.23		1.02			
	BSK1-1	*LOC_Os03g04050*					-1.01				
	BSK1-2	*LOC_Os10g39670*									-1.00
	BSK2	*LOC_Os10g42110*	-2.85		-3.91	-3.06				1.39	-2.74
	BSK3	*LOC_Os04g58750*									
	BSK4	*LOC_Os03g61010*	-4.60	-1.64	-5.29	-3.49				1.23	-2.66
	PPKL1	*LOC_Os03g44500*									-1.17
	PPKL2	*LOC_Os05g05240*		-1.06							
	PPKL3	*LOC_Os12g42310*									
	GSK1	*LOC_Os01g10840*	-1.26	-1.24	-1.67						
	BZR1	*LOC_Os07g39220*			-1.39	-1.87		-1.69			-3.79
	BZR2	*LOC_Os01g10610*						-1.49	-1.06	-1.18	-2.37
	BZR3	*LOC_Os06g35900*				-1.38					
	BZR4	*LOC_Os02g13900*			-2.37						-1.89

Supplemental Figure S10. Differentially expressed BR-related genes in different tissues of different plants. (Supports Figure 8)

Values of log2FC (fold change) of each gene in transcriptome analyses compared to ZH11 were shown, and the background was decorated according to the change direction and amplitude.

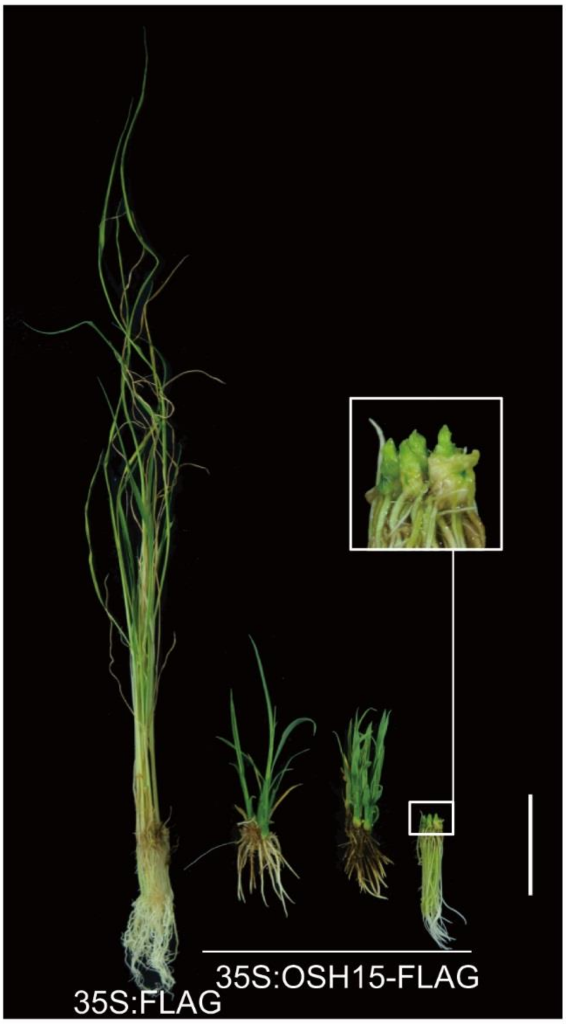

Supplemental Figure S11. Phenotypes of OSH15-overexpression plants. (Supports Figure 8)
A transgenic line with introduction of an empty vector (35S:FLAG) (left) was shown as control. White box shows the close view of a severe line with strong defect of leaf development. Bar = 4 cm.

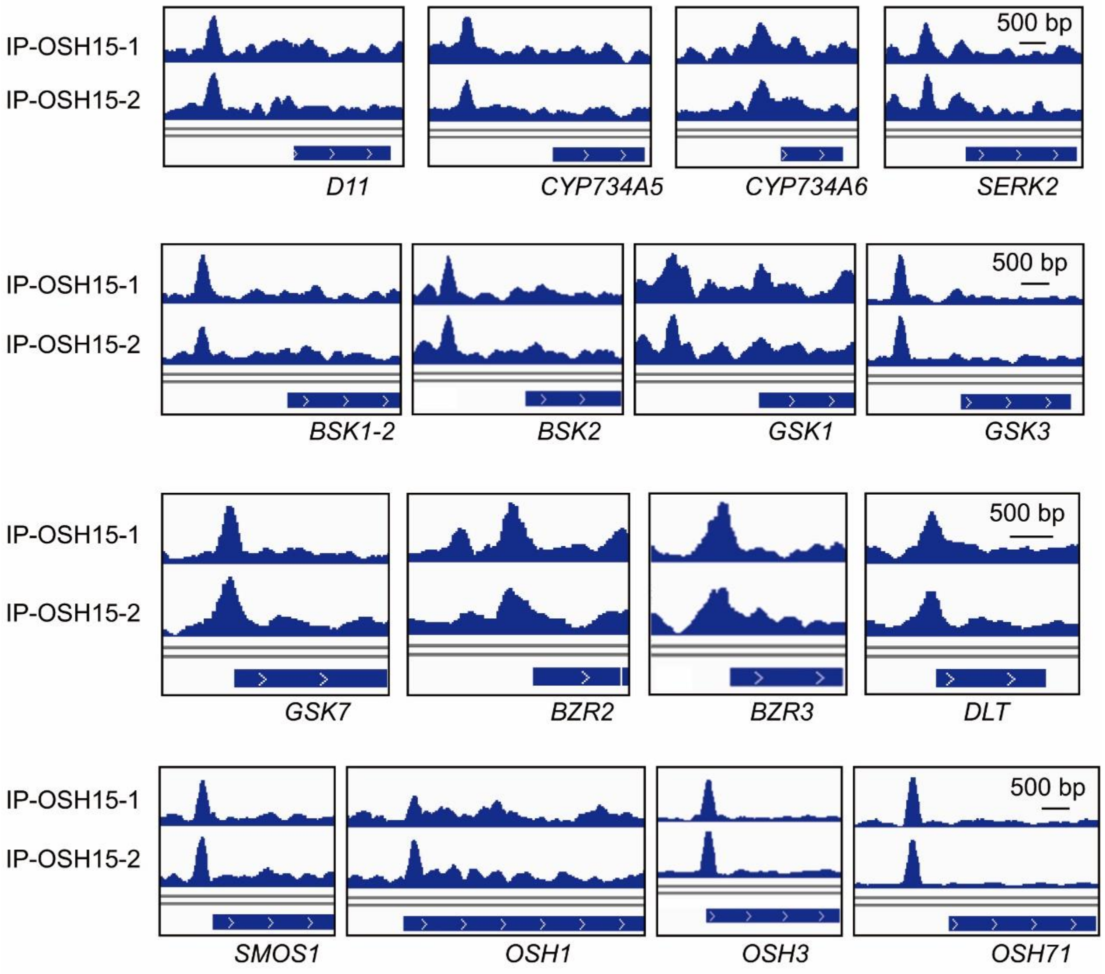

Supplemental Figure S12. Visualization of the binding peaks in ChIP-seq. Two replicates were included and shown. (Supports Figure 8)

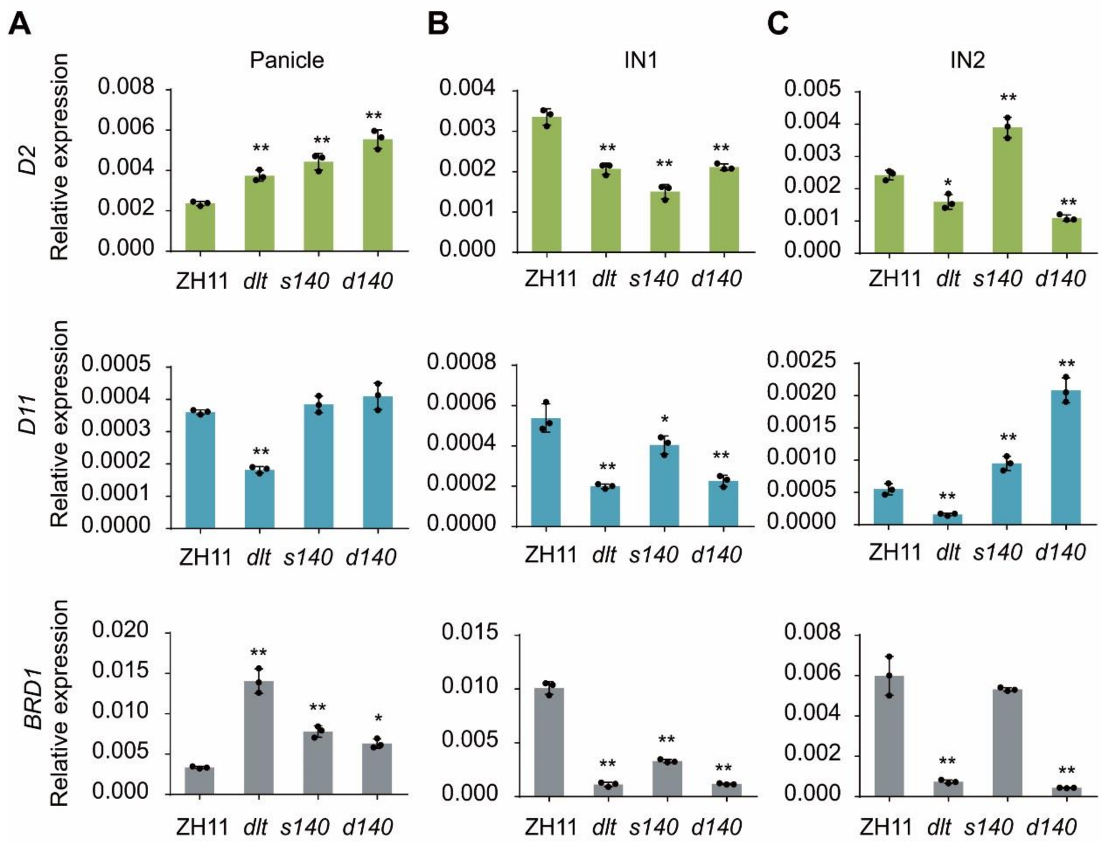

Supplemental Figure S13. Expression of BR biosynthetic genes in different tissues. (Supports Figure 9)

Panicle (A), IN1 (B) and IN2 (C) of ZH11, *dlt*, *s140* and *d140* were used for RT-quantitative PCR analysis. Data are shown as means ± SD (*n* = 3). Asterisks indicate significant difference compared with ZH11, with *P < 0.05 and **P < 0.01 by Student's two-sided *t*-test.

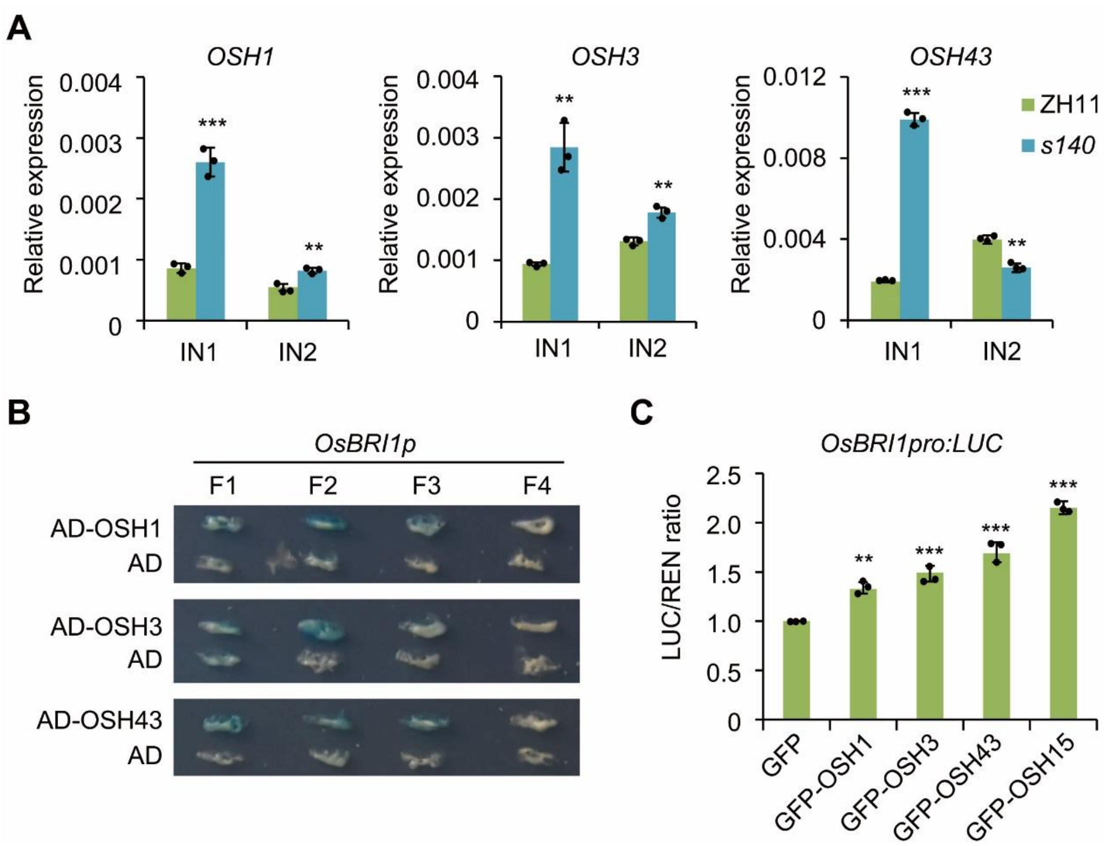

Supplemental Figure S14. OSH homologs might compensate for OSH15 defection in IN1. (Supports Discussion)

(A) Expression of OSH1, OSH3, and OSH43 in IN1 and IN2 of ZH11 and *s140*. Asterisks indicate significant difference compared with ZH11, with **P < 0.01 and ***P < 0.001 by Student's two-sided *t*-test.

(B) Yeast one-hybrid analysis testing the binding of OSH members on *OsBRI1* promoter.

(C) Effect of OSH members on *OsBRI1* promoter activity using luciferase as reporter. Data are shown as means ± SD (*n* = 3). Asterisks indicate significant difference compared with the sole GFP, with **P < 0.01 and ***P < 0.001 by Student's two-sided t-test.

Supplemental Table S1. Hormone quantification data.

Tissue	Sample	CS (Means ± SD, ng·g^{-1} F.W.)	Change (%, /ZH11)	P value (*t*-test)
**Seedling**	ZH11	0.19 ± 0.01	N.A.	N.A.
	dlt	0.24 ± 0.01	+30.70	1.95E-03
	s140	0.23 ± 0.00	+25.31	3.02E-03
	d140	0.42 ± 0.09	+123.70	1.31E-02
**Panicle**	ZH11	0.75 ± 0.11	N.A.	N.A.
	dlt	0.78 ± 0.08	+4.45	7.00E-01
	s140	1.07 ± 0.04	+42.73	9.65E-03
	d140	1.28 ± 0.21	+70.34	1.96E-02
**IN1**	ZH11	0.17 ± 0.02	N.A.	N.A.
	dlt	0.15 ± 0.02	–13.73	2.34E-01
	s140	0.06 ± 0.00	–64.71	6.78E-04
	d140	0.03 ± 0.00	–82.35	2.66E-04
**IN2**	ZH11	0.08 ± 0.00	N.A.	N.A.
	dlt	0.06 ± 0.01	–13.33	4.74E-02
	s140	0.05 ± 0.00	–33.33	1.32E-03
	d140	0.05 ± 0.01	–26.67	1.61E-02

F.W., fresh weight; N.A., not applicable. *n* = 3 for all measurements. + indicates increase, and – indicates decrease. P values are shown using scientific notation, with the index number following E (exponent).

Supplemental Table S2. Statistical analysis of seedling height, IN1 length, IN2 length, and tiller number in different plants.

**Tissue**	**Plant**	**Value (Means ± SD)**	**Change (%, /ZH11)**	**P value (*t*-test)**
**Seedling height (cm)**	ZH11	24.91 ± 1.34	N.A.	N.A.
	dlt	19.69 ± 0.59	–20.96	1.39E-12
	s140	26.2 ± 0.76	+5.17	2.96E-3
	d140	20.13 ± 1.02	–19.20	1.59E-10
	s76	22.25 ± 1.13	–10.70	6.08E-06
	d76	13.25 ± 0.55	–46.80	1.59E-20
**IN1 (cm)**	ZH11	38 ± 0.82	N.A.	N.A.
	dlt	26.62 ± 0.75	–29.93	4.36E-07
	s140	31 ± 1.41	–18.42	6.92E-05
	d140	21.32 ± 0.97	–43.89	1.10E-08
	s76	27.35 ± 0.77	–28.03	6.98E-07
	d76	22.3 ± 0.5	–41.32	1.74E-09
**IN2 (cm)**	ZH11	21 ± 0.41	N.A.	N.A.
	dlt	13.5 ± 0.51	–35.71	2.23E-07
	s140	4.2 ± 0.68	–80.00	5.72E-09
	d140	3.6 ± 0.39	–82.86	6.20E-10
	s76	3.45 ± 0.42	–83.57	7.27E-10
	d76	3.44 ± 0.40	–83.62	2.82E-11
**Tiller number**	ZH11	12.5 ± 3.38	N.A.	N.A.
	dlt	6.29 ± 1.38	–49.71	2.84E-04
	s140	21.2 ± 2.68	+69.60	2.54E-04
	d140	9.86 ± 2.34	–21.14	5.34E-02
	s76	19.6 ± 1.14	+56.80	4.70E-04
	d76	8.67 ± 1.21	–30.67	1.10E-02

N.A., not applicable. n = 13 for measurement of seedling height, = 12 for others. + indicates increase, and – indicates decrease. P values are shown using scientific notation, with the index number following E (exponent).

Supplemental Table S3. Information for vector construction.

**Name**	**Forward and Reverse primer sequences (5'-3')**	**Empty vector**
OSH15-AD	F: GGAGGCCAGTGAATTCATGGATCAGAGCTTTG	pGADT7
	R: CGAGCTCGATGGATCCTCACGAACCGAGGCGG	
OSH15-BD	F: CATGGAGGCCGAATTCATGGATCAGAGCTTTG	pGBKT7
	R: GCAGGTCGACGGATCCTCACGAACCGAGGCGG	
DLT-AD	F: GGAGGCCAGTGAATTCATGTTGGCGGGTTGCTC	pGADT7
	R: CGAGCTCGATGGATCCTTAGCTTTGCTGAGAATG	
GSK2-AD	F: GGAGGCCAGTGAATTCCAGCTTTGCCACATGGAC	pGADT7
	R: CGAGCTCGATGGATCCTTAGCTCCCAGTATTGAA	
DLT(C)-AD	F: CATATGCTGGAGCTGGTGCGCGCG	pGADT7
	R: CTCGAGTTAGCTTTGCTGAGAATGTGATGCTGTTG	
NLuc-OSH15	F: TCGGTACCCGGGATCCATGGATCAGAGCTTTG	pCAMBIA1300-35S-Nluc
	R: ACGAGATCTGGTCGACCGAACCGAGGCGGTAC	
DLT-CLuc	F: AGCGGTACCCGGGATCC ATGTTGGCGGGTTGCT	pCAMBIA1300-35S-CLuc
	R: AGCTCTGCAGGTCGACTTAGCTTTGCTGAGAATG	
OSH15-GFP	F: GGGTACCCGGGGATCCATGGATCAGAGCTTTG	pCAMBIA1300-35S-eGFP
	R: TGGTACTAGTGTCGACCGAACCGAGGCGGTAC	
DLT-FLAG	F: GATGATAAGGGCGGTACCATGTTGGCGGGTTGCT	pCAMBIA1300-35S-FLAG
	R: AGGCTACGTAGGATCCTTAGCTTTGCTGAGAATG	
OSH15-FLAG	F: GATGATAAGGGCGGTACCATGGATCAGAGCTTTG	pCAMBIA1300-35S-FLAG
	R: AGGCTACGTAGGATCCTCACGAACCGAGGCGG	
OSH15-PB42AD	F: TGCCTCTCCCGAATTCATGGATCAGAGCTTTG	PB42AD
	R: CGAGTCGGCCGAATTCCGAACCGAGGCGGTAC	
DLT-PB42AD	F: TGCCTCTCCCGAATTCATGTTGGCGGGTTGCTC	PB42AD
	R: CGAGTCGGCCGAATTCGCTTTGCTGAGAATG	

F1-Placzi2u	F: ATCTGTCGACCTCGAGCTCGTTTTACATAAAACA<br>R: GAGCACATGCCTCGAGCCTGACAGGCATGAGATC	Placzi2u
F2-Placzi2u	F: ATCTGTCGACCTCGAGGATCTCATGCCTGTCAGG<br>R: GAGCACATGCCTCGAGGGTGCTCCGCTTCCCCA	Placzi2u
F3-Placzi2u	F: ATCTGTCGACCTCGAGCGTGGGCATATTGGGGAA<br>R: GAGCACATGCCTCGAGGCATGACACACCAGTGA	Placzi2u
F4-Placzi2u	F: ATCTGTCGACCTCGAGTACAGCTGTTAAGGCCA<br>R: GAGCACATGCCTCGAGGTACGAGCGAGCTCACT	Placzi2u
BRI1P-LUC	F: CGGTATCGATAAGCTTCTCGTTTTACATAAAACA<br>R: TGTAAAAATAAAGCTTGTACGAGCGAGCTCAC	pGREEN-LUC
OSH1-PB42AD	F: TGCCTCTCCCGAATTCATGGAGGAGATCTCCCA<br>R: TCCAAAGCTTCTCGAGGAAGAGCCGGAGGAAAG	PB42AD
OSH3-PB42AD	F: TGCCTCTCCCGAATTCATGGAGGAGCTTGAAGG<br>R: TCCAAAGCTTCTCGAGTGCGCGCCACACGCCA	PB42AD
OSH43-PB42AD	F: TGCCTCTCCCGAATTCATGGAGAGCTTCGCCAG<br>R: TCCAAAGCTTCTCGAGAGACCCGAGCCGGTAC	PB42AD
BRI1genome-LUC	F: CGGTATCGATAAGCTTCTCGTTTTACATAAAACA<br>R: TGTAAAAATAAAGCTTATCCTTCTCCTCCTTGG	pGREEN-LUC
OSH15P-GUS	F: TGATTACGCCAAGCTTGAGCCGAAGGGAGTTT<br>R: GACGGCCAGTGAATTCCAAAACACACACAAGA	2391Z

Restriction enzyme sites used for vector construction are underlined.

Supplemental Table S4. Primers used for RT-qPCR.

Gene Name	Forward 5’-3’	Reverse 5’-3’
UBQ	GAGCCTCTGTTCGTCAAGTA	ACTCGATGGTCCATTAAACC
OSH15	AGAGGAAACGGCACTGGAA	AGCAGCATTCTGTGGGTG
DLT	TGCGGATACTCAACGCCATCA	ACTCGCCGACTCCGGTGATC
D2	CCTTTTGGTGGTGGGCAGAG	TGGGGAAGTTGACGATGTGGT
D11	TTGGGTCATGGCATGGCAAGAGCAAGGA	TTGTTGCTGGAGCCAGCATTCCTCCTCT
BRD1	GAGGTGGCTGGAGAAGAACAT	ATTTCTACGGTGCCTACTTCCT
BRI1	CAGCTACTTGGCTATCTTGAAGCTCAGC	CCATTCTTGTTGAAGGTGTACTCCGTGC
LOC_Os01g58850	ACCATTCCTTCCGGCCATTCTG	ACGACATTGAGCAACCTGAACCG
LOC_Os02g41954	AAGGGTGCAGGAAGACGTCAAG	TGAATTTGTGTGCGCCTCATACG
LOC_Os02g52670	ACGTCCACTCCTCCATCACAAC	AGCATCATGATCTCCCGTAGGC
LOC_Os05g07010	AGATGACCTGGGATTCGCTCAG	TCTTAAGCACCAGCAAGATCGTC
LOC_Os06g03670	AGCTACTGATGATCGCGAGTTGG	AGGAGGAGCAAAGCTGGTTGAG
LOC_Os07g05940	TTCGCCGTCACCGAGAACTATG	CTGGAGCTTGAACACGATCTGC
LOC_Os08g04800	GAATGCGGGTTCTGGAACCTCTAC	TTTGTCAGTAGCCTCCGTACCTC
LOC_Os08g36920	ACTCACATGACCAACCGGATCTC	GCCGTCGAATCGGATCATGTACTC
LOC_Os11g10590	TGTCGTCTCCGCAGAGTCAAAG	CGCAGCAATCCATAGGACGAAAC
LOC_Os12g05440	ACCTGAAGCGGCTGAACTACAC	CGCAGAACTTGGAGTCGAACTG
ChIP-P1	GCATGGATAGTTTAGGTT	ATTGTGTACGCATGTTAG
ChIP-P2	CGGCTCGTTTCCACCCCT	TGCTCCGCTTCCCCAATA
ChIP-P3	ACCCCACTTCGGAAAT	ATTTCACGCCTGTGGT
ChIP-P4	CGGGGGGGCTGCGAT	TTCAGTCTTACCCCTAT
ChIP-P5	CAAGTCCTCGGTAACA	GGAGGCGGACGCAAGA
ChIP-P6	TGGGAAGATGAATGTTG	CAGGTCGGATGCTGGTA
OSH1	GCGGCAAGGCGAAGGA	TCTGGCAGTCGAGGTAGGC
OSH3	GAAGAGGAAGAAAGGGAAG	CGCTGGTTGATGAAACAGT
OSH43	GGAGTTCTTCAGCAGGGT	GTCCTCTTGGAGAACACCT
